# Access, acceptance and adherence to cancer prehabilitation: a mixed-methods systematic review

**DOI:** 10.1007/s11764-024-01605-3

**Published:** 2024-05-06

**Authors:** Tessa Watts, Nicholas Courtier, Sarah Fry, Nichola Gale, Elizabeth Gillen, Grace McCutchan, Manasi Patil, Tracy Rees, Dominic Roche, Sally Wheelwright, Jane Hopkinson

**Affiliations:** 1 Cardiff University, Cardiff, UK; 2 University of Sussex, Brighton, UK

**Keywords:** Cancer, Prehabilitation, Systematic review, Access, Barriers, Facilitators

## Abstract

**Purpose:**

The purpose of this systematic review is to better understand access to, acceptance of and adherence to cancer prehabilitation.

**Methods:**

MEDLINE, CINAHL, PsychINFO, Embase, Physiotherapy Evidence Database, ProQuest Medical Library, Cochrane Library, Web of Science and grey literature were systematically searched for quantitative, qualitative and mixed-methods studies published in English between January 2017 and June 2023. Screening, data extraction and critical appraisal were conducted by two reviewers independently using Covidence™ systematic review software. Data were analysed and synthesised thematically to address the question ‘What do we know about access, acceptance and adherence to cancer prehabilitation, particularly among socially deprived and minority ethnic groups?’

The protocol is published on PROSPERO CRD42023403776

**Results:**

Searches identified 11,715 records, and 56 studies of variable methodological quality were included: 32 quantitative, 15 qualitative and nine mixed-methods. Analysis identified facilitators and barriers at individual and structural levels, and with interpersonal connections important for prehabilitation access, acceptance and adherence. No study reported analysis of facilitators and barriers to prehabilitation specific to people from ethnic minority communities. One study described health literacy as a barrier to access for people from socioeconomically deprived communities.

**Conclusions:**

There is limited empirical research of barriers and facilitators to inform improvement in equity of access to cancer prehabilitation.

**Implications for Cancer Survivors:**

To enhance the inclusivity of cancer prehabilitation, adjustments may be needed to accommodate individual characteristics and attention given to structural factors, such as staff training. Interpersonal connections are proposed as a fundamental ingredient for successful prehabilitation.

**Supplementary Information:**

The online version contains supplementary material available at 10.1007/s11764-024-01605-3.

## Introduction

Prehabilitation is a core component of supportive care for health and well-being during cancer survivorship. It aims to improve cancer treatment outcomes and long-term health by preparing people awaiting cancer treatments, not only surgery, through support for physical activity, nutrition and emotional well-being either alone or in combination, and from the point of diagnosis [1]. Growing international evidence indicates that, in specific cancers, engagement with either uni or multimodal prehabilitation interventions can improve individuals’ pre-treatment functional capacity [2, 3], reduce treatment-related complications [4–6], ease anxiety [7] and enhance post-treatment recovery [8, 9]. As the evidence base develops and momentum for prehabilitation grows, the need to embed prehabilitation as the standard of care across different cancers has been recognised [10–12]. In some regions, multimodal prehabilitation is now offered as the standard of care in certain cancers, particularly lung [13] and colorectal [14].

Internationally, there are persistent health disparities following cancer treatment. Treatment and survival outcomes are poor among people from socioeconomically deprived communities and some minority ethnic groups compared to socioeconomically advantaged and majority groups [15–17]. To ease the overall social and economic impact of cancer on individuals and society, and to reduce the societal and healthcare costs of suboptimal treatment outcomes, it is important to identify the facilitators of and barriers to individuals’ engagement with interventions. People from socioeconomically deprived communities and some minority ethnic groups are known to be underserved in prehabilitation interventions [1, 18]. Accordingly, to better understand reasons for informed action, this mixed-methods systematic review aims to identify, critically appraise and synthesise international empirical evidence of the facilitators of and barriers to access, acceptance  and adherence of cancer prehabilitation. For this review, prehabilitation is defined as proactive and preventative for all cancer treatments (not only surgery and including neoadjuvant) and includes interventions to support physical activity, nutritional intake or psychological well-being, alone or together, carried out at any time before a course of treatment begins.

### Review question

What is known about access, acceptance and adherence to cancer prehabilitation, particularly among socially deprived and minority ethnic groups?

## Methods

The systematic review was informed by the Joanna Briggs Institute (JBI) mixed-methods systematic reviews (MMSR) methodology [19]. A convergent, integrated approach to data synthesis and integration was adopted [19, 20]. The review was registered in PROSPERO CRD42023403776) on 3 March 2023 and is reported in accordance with the Preferred Reporting Items for Systematic Review and Meta-Analysis (PRISMA) guidelines [21]. Ethical approval was not required.

### Database searches

In collaboration with a specialist health service systematic review librarian, the search strategy was developed using medical subject headings (MeSH) and keywords including and relating to cancer, prehabilitation, inequity, inequality, socioeconomic deprivation, ethnic groups and health services accessibility, and then tested and refined. The electronic databases Ovid SP MEDLINE, CINAHL via EBSCO host, PsycINFO, Ovid SP EMBASE, Ovid Emcare, Allied and Complementary Medicine (AMED), Physiotherapy Evidence Database (PEDRo) and Cochrane Central were systematically searched by EG for studies published in English between January 2017 and May 2023. The search strategy was tailored for each database and detailed in online resource (Supplementary information 1). Supplementary searches of grey literature using the Overton, Dimensions and Proquest dissertation and theses databases (PQDT), and relevant organisational websites were conducted. Reference lists of papers retrieved for full review were scrutinised for potentially useful papers not identified through the database searches.

### Selection criteria

The PICO framework was used to guide inclusion criteria on population (P), Intervention (I), comparators (C) and outcomes (O) and context (Co). It enabled identification of primary qualitative, quantitative and mixed-methods research studies about prehabilitation, published in peer-reviewed journals. Eligibility criteria were used during study selection to screen this body of literature for empirical data about barriers and facilitators of prehabilitation. Non-empirical, opinion pieces, theoretical and methodological articles, reviews and editorials were excluded, as were studies involving children, adolescents and focusing on end-of-life care.

### Study selection

All search results were stored in Endnote™. Following deduplication, results were imported into Covidence™ systematic review management software. For study selection, standardised systematic review methods [22] were used. All project team members were involved in study screening and selection. Firstly, two reviewers independently screened all returned titles and abstracts. Based on eligibility and relevance, these were sifted into ‘yes’, ‘no’ or ‘maybe’ categories. Disagreements were resolved by a third reviewer. Where a definite decision could not be made, full text was retrieved and assessed. Secondly, full text of all potentially relevant abstracts was retrieved and independently assessed for inclusion by two reviewers against the eligibility criteria. Arbitration by an independent reviewer in the event of disagreement was not required at this stage. Reasons for exclusion at full text review were recorded.

### Quality assessment

Two reviewers independently assessed the quality of included studies via Covidence ™ using the Mixed Methods Appraisal Tool (MMAT) version 18 [23]. The MMAT was constructed specifically for quality appraisal in mixed studies reviews and is widely used [23, 24]. Within a single tool, Version 18 of the MMAT can be used to appraise the methodological quality of five broad categories of study design, namely qualitative, randomised controlled trials, non-randomised, quantitative descriptive and mixed methods studies. The MMAT comprises two screening questions to establish whether or not the quality appraisal should proceed and 25 core questions: five criteria which mostly relate to the appropriateness of study design and approaches to sampling, data collection and analysis relevant to each of the five study designs [23]. Each criterion is assessed as being met (Yes) or not (No). There is also scope to indicate uncertainty. A third reviewer independently moderated all quality assessments for accuracy.

### Data extraction

Two reviewers independently extracted data systematically via Covidence™ using an adapted, piloted JBI mixed-methods data extraction form. Information extracted included study author, aim, year and country of publication, setting, intervention type, design, sample, data collection, analysis, data relating to prehabilitation facilitators and barriers and, as relevant, data on intervention for support of access, acceptance or adherence to prehabilitation. A third reviewer cross-checked the data extraction tables independently for accuracy and completeness.

### Data synthesis and integration

All extracted findings were imported into Microsoft Excel. Quantitative data were ‘qualitised’ into textual descriptions of quantitative results to enable assimilation with qualitative data [25]. To analyse and synthesise all findings, thematic synthesis [26, 27] was used. Thematic analysis is an established process involving the identification and development of patterns and analytic themes in primary research data. Two reviewers coded the findings and then grouped related codes into preliminary descriptive themes which captured patterns across the data describing barriers to and facilitators of cancer prehabilitation [26]. Preliminary themes were discussed with a third reviewer. Themes were then further combined and synthesised to generate three overarching analytical themes relative to the review question [26].

## Results

**Fig. 1:**
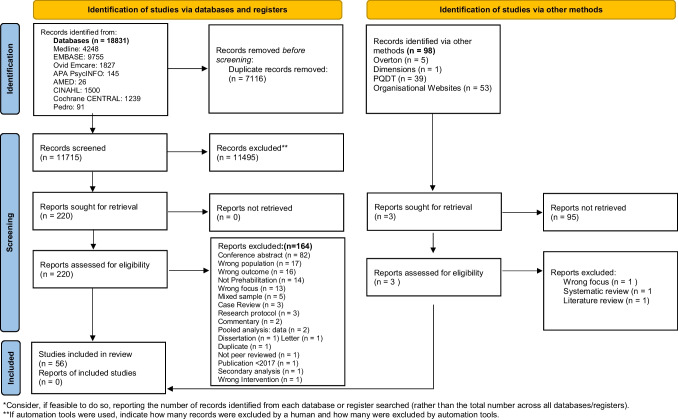
PRISMA 2020 flow diagram for new systematic reviews which included searches of databases, registers and other sources. *Consider, if feasible to do so, reporting the number of records identified from each database or register searched (rather than the total number across all databases/registers). **If automation tools were used, indicate how many records were excluded by a human and how many were excluded by automation tools. From: Page MJ, McKenzie JE, Bossuyt PM, Boutron I, Hoffmann TC, Mulrow CD, et al. The PRISMA 2020 statement: an updated guideline for reporting systematic reviews. BMJ 2021;372:n71. 10.1136/bmj.n71. For more information, visit: http://www.prisma-statement.org/

Figure 1 shows the PRISMA flow chart of search results. Following the first and second round screening, 56 papers published between 2017 and 2023 were included: 33 quantitative; 14 qualitative and nine mixed methods.

A synopsis of study characteristics and the quality appraisal outcomes is found in Table 1. Brief narrative summaries of the included papers’ findings of relevance to the review question, namely access, acceptance and adherence of prehabilitation interventions, are provided in the online supplementary information (supplementary information 2).

**Table 1:** Characteristics of studies

Study author Year Country	Aim	Design	Setting/prehabilitation intervention	Study participants	Sample size	MMAT Score/Category
Quantitative studies						
Prepare ABC Trial Collaborative [28] 2021 United Kingdom	To confirm feasibility of site set-up and patient recruitment, acceptability of the interventions and patient adherence to hospital-supervised and home-supported exercise.	Pilot randomised controlled trial	Hospital and home based exercise prehabilitation	People with colorectal cancer	200 **Hospital supervised** *n=*68 69% Male Mean age 67.6 [range 35–86] **Home supported** *n =*69 67% Male Mean age 66.7 [range 39–84] **Treatment as usual** *n=* 63 68% male Mean age 69.1 [range 53–85] Ethnicity not reported. Socio-economic status not reported.	80% RCT
Argudo [29] 2021 Spain	To investigate the feasibility and tolerability of a 5-week preoperative high-intensity interval training program after NAT, and to assess the potential effects of the training protocol on exercise capacity, muscle function, and health-related quality of life (HRQL)	Prospective pilot intervention study	Blended exercise prehabilitation	People with locally advanced upper gastrointestinal cancers.	33 79% Male Mean age 65 [SD 12] Ethnicity not reported. Socio-economic status not reported.	100% Quantitative Non-Randomised
Bradley 2023 [13] United Kingdom	Examine the feasibility, uptake, participation, and clinical outcomes from the Greater Manchester Prehab4Cancer (P4C) programme for lung cancer patients with planned surgical resection.	Feasibility study	Regional community based multimodal prehabilitation programme	People with lung cancer	377 Female 52.3% Mean age 72 [IQR 66–77] Ethnicity not reported. Socio-economic status not reported.	80% Quantitative Descriptive
Burden 2017 [30] United Kingdom	To determine if pre-operative ONS with dietary advice, compared with dietary advice only, can reduce post-operative infections in people prior to surgical re-section for colorectal cancer who have previously lost weight	Randomised controlled trial	Hospital based nutritional intervention	People with colorectal cancer	101 **Control and dietary advice only** *n=*46 Male 70% Mean age 68.9 [SD 11.49] **Occupation** Professional *n=* 13 Skilled *n=* 16 Unskilled *n=* 14 Unemployed *n=* 0 Missing *n=* 3 **Intervention ONS and dietary advice** *n=*55 Male 64% Mean age 70.5 [SD 11.66] **Occupation** Professional *n=* 19 Skilled *n=* 19 Unskilled *n=* 12 Unemployed *n=* 2 Missing *n=* 3 Ethnicity not reported. Socio-economic status not fully reported	80% RCT
Catho 2021 [31] France	To identify determinants of non-completion of an H-RP and the factors associated with medical events occurring 30 days after hospital discharge.	Prospective observational study	Home-based Multimodal prehabilitation	People with proven or suspected non-small cell lung cancer	50 Male 81% Mean age 69 [range 60–74] Ethnicity not reported. Socio-economic status not reported.	80% Quantitative Descriptive
Crowe 2022 [32] Australia	To evaluate the impact of a new multidisciplinary allied health prehabilitation service in haematologic cancer patients receiving high-dose chemotherapy with autologous stem cell transplant (AuSCT).	Retrospective cohort study	Hospital based multimodal prehabilitation	People with haematological cancers	121 Male 52.9% Mean Age 64.0 [56.0–69.0] Ethnicity not reported. Socio-economic status not reported.	80% Quantitative Descriptive
Deftereos 2021 [33] Australia	To determine the type and frequency of preoperative dietetics intervention and nutrition support received, factors associated with receipt of preoperative dietetics intervention.	Prevalence study	Hospital based preoperative dietetic and nutritional prehabilitation	People with upper gastrointestinal tract cancers	200 Male 58.5% Mean age 67 [SD10] Ethnicity not reported. Socio-economic status not reported.	80% Quantitative Descriptive
Drummond 2022 [34] Canada	To document the implementation of a multi-modal teleprehabilitation for cancer patients undergoing elective thoracic and abdominal cancer resection surgery and patients' experience of the program	Pilot cohort study (retrospective)	Multimodal teleprehabilitation programme	People with upper gastro-intestinal, lung and colorectal cancers	10 Male 80% Mean age 68 [range 52–88] Ethnicity not reported. Socio-economic status not reported.	80% Quantitative Descriptive
Ferreira 2021 [35] Canada	To assess the feasibility of delivering a novel four-week multimodal prehabilitation intervention combining a mixed-nutrient supplement with structured exercise training and relaxation-strategies for patients with lung cancer awaiting surgical resection	Randomised controlled trial	Blended multimodal prehabilitation programme	People with lung cancer	34 **Prehabilitation** *n=*24 Male 54% Mean age 67 [range 63.3–72] **Control** *n=*10 Male 50% Mean age 69 [range 66.8–73.3] Ethnicity not reported. Socio-economic status not reported.	80% RCT
Franssen 2022 [36] Netherlands	To investigate whether a home-based and tele-monitored prehabilitation program (tele-prehabilitation) is feasible in high-risk patients scheduled for colorectal cancer surgery and to evaluate patient experiences and changes in pre-operative aerobic fitness before and after the tele-prehabilitation program.	Feasibility study Quantitative Descriptive	Bimodal (Exercise and nutrition) tele - prehabilitation	People with colorectal cancer	11 Male 55% Age 74 [range 68–78] Ethnicity not reported. Socio-economic status not reported.	60% Quantitative Descriptive.
Halliday 2021 [37] United Kingdom	To establish whether adherence to a personalised exercise prescription and the amount of physical activity (PA) completed during prehabilitation are related to cardiorespiratory fitness and the incidence of post-operative pneumonia	Cohort study	Home based exercise prehabilitation	People with oesophageal cancer	67 Age 66 [SD 9.7] Gender not reported. Ethnicity not reported. Socio-economic status not reported.	80% Quantitative Descriptive.
Karlsson 2019 [38] Sweden	To evaluate the feasibility of a preoperative, supervised home-based physical exercise program at a high level of estimated exertion, in older people undergoing colorectal cancer surgery in Sweden: Can it be done? Should it be done? And if so, how?	Randomised controlled trial	Home based exercise prehabilitation	Older people with colorectal cancer Instructors	23 patients **Intervention** *n=*11 Male 40% Median age 83.5 [range 76–85] **Standard care** *n=*12 Male 36% Median age 74.0 [range 73–76] Ethnicity not reported. Socio-economic status not reported. **Instructors** *n=*6	80% RCT
Lawson 2021 [39] Canada	Feasibility and preliminary outcome data on the effects of a 4-week multimodal prehabilitation intervention on muscle characteristics and dietary intake of surgical lung cancer patients.	Randomised controlled Trial	University health centre. Blended multimodal prehabilitation	People with lung cancer	34 **Intervention** *n=*24 Male 54% Median age 67 [range 63–72] **Standard care** *n=*10 Male 50% Median age 69 [67–73]	60% RCT
Machado 2023 [40] Portugal	To determine the feasibility of a homebased exercise program (HBEP) in lung cancer patients undergoing surgical treatment	Prospective, single arm, two site trial	Home based exercise prehabilitation	People with suspected or confirmed lung cancer scheduled for surgery	15 Male 60% Mean age 67.5 [SD 8.1] **Educational level** <10 years 66.7% >10 years 33.3% Ethnicity not reported.	60% Quantitative Descriptive
Minnella 2021 [41] Canada	To assess the safety and the feasibility of personalized, stepped-approach prehabilitation care in the context of ERP for elective pulmonary cancer surgery.	Feasibility study Quantitative non-randomized	Home and hospital multimodal prehabilitation	People with lung cancer	81 **Prehabilitation** *n=* 45 Male 62% Age > 75 33% **Standard care** *n=*36 Male 42% Age > 75 19% Ethnicity not reported. Socioeconomic status not reported.	60% Quantitative non-randomised
Moorthy 2023 [42] United Kingdom	To establish the feasibility of delivering a digital prehabilitation service.	Feasibility study Quantitative Descriptive	Digital multimodal prehabilitation	People with upper gastrointestinal cancer	57 **Digital programme** *n=*31 Male 84% Mean age 67.4 [SD 8.9] **In -person programme** *n=* 26 Male 65% Mean age 65 [SD 10.1] Ethnicity not reported. Socioeconomic status not reported.	40% Quantitative Descriptive
Naito 2019 [43] Japan	To test the feasibility of the early induction of new multimodal interventions specific for elderly patients with advanced non-small cell or pancreatic cancer	Multi-centre, prospective single arm	Blended bimodal (exercise and nutrition) prehabilitation	Older people with pancreatic and lung cancers	30 Age 75 [70–84] Gender Ratio women:men 10:20 Ethnicity not reported. Socioeconomic status not reported.	80% Quantitative Descriptive
Paynter 2017 [44] Australia	To determine patient acceptance of a pre-operative immunonutrition supplement protocol and to compare post-operative outcomes pre- and post-implementation of the protocol	Single Centre retrospective audit	Hospital based nutritional prehabilitation	People with upper gastrointestinal cancers	74 **Pre Implementation** *n=* 36 Male 56% Age 63.61 [SD 1.69] **Post implementation** *n=* 38 Male 61% Age 63.76 [SD 2.03] Ethnicity not reported. Socioeconomic status not reported.	80% Quantitative Descriptive
Piraux 2020 [45] Belgium	To assess the feasibility and the preliminary effects of a tele-prehabilitation program in esophagogastric cancer patients requiring surgery.	Feasibility study Quantitative descriptive	Exercise teleprehabilitation	People with upper gastrointestinal tract cancers	23 Male 70% Age 61.7 [SD 10.6] Ethnicity not reported. Socioeconomic status not reported.	60% Quantitative Descriptive
Qin 2022 [46] China	What is the association between health literacy and enhanced recovery after surgery (ERAS) adherence and postoperative outcomes in patients undergoing colorectal surgery?	Prospective cohort study	Hospital based ERAS intervention	People with colorectal cancer	865 **Full cohort** **High HL** *n=*329 Male 61.1% Age 61 [IQR 53.0–68.0] **Education** < Middle school 8.8% Middle school 36.5% High School 31.9% > High School 22.8% **Household income** Low 29.8% Moderate 39.8% High 30.4% **Low HL** *n=* 536 Male 59.7% Age 66 [IQR 58.0–73.0] **Education** < Middle school 42% Middle school 39.2% High School 13.2% >High School 5.6% **Household income** Low 42.2% Moderate 38.1% High 19.8% **Propensity score–matched cohort** *n=*480 **High HL** *n=*240 Male 59.2% Age 62.5 [IQR 55.3–69.0] **Education** < Middle school 12.1% Middle school 47.9% High School 29.2% > High School 10.8% **Household income** Low 34.6% Moderate 37.1% High 28.3% **Low HL** *n=* 240 Male 59.2% 63.5 [IQR 54.0–70.0] **Education** < Middle school 13.3% Middle school 45.8% High School 28.3% > High School 12.5% **Household income** Low 35.4% Moderate 36.7% High 27.9% Ethnicity not reported	80% Quantitative non-randomised
Rupnik 2020 [47] Slovenia	To investigate the feasibility and safety of a multimodal intervention programme with partially supervised exercise training combined with nutritional support prior to HSCT.	Single arm pilot study	Blended bimodal (Exercise and nutritional support) prehabilitation	People with haematological cancers	28 Male 64% Mean age 59.4 [SD 8.2] Ethnicity not reported. Socioeconomic status not reported.	80% Quantitative Descriptive
Santa Mina 2018 [48] Canada	To assess the feasibility and effect of a personalised, home-based prehabilitation intervention on clinically-relevant outcomes in radical prostatectomy patients.	Multicentre RCT	Home based exercise prehabilitation	Men with prostate cancer	86 **Prehabilitation intervention** *n=*44 Age 61.2 [SD 8.0] **Ethnicity** White/Caucasian 68% Black/Afro-Caribbean/African 14% Ashkenazi Jewish 2% East and South Asian 5% South East Asian 2% Other 7% Missing 0% **Annual income** <$40,000 11% $40,000–$80,000 59% > $80,000 27% Missing 2% **Education** < High school 9% High school graduate 18% Community college 14% University undergraduate or graduate degree 50% Other 7% Missing 2% **Employment status** Full-time 41% Unemployed 2% Part-time 23% Retired 32% Missing 2% **Control** *n=*42 62.2 [SD 6.9] **Ethnicity** White/Caucasian 71% Black/Afro-Caribbean/African 12% Ashkenazi Jewish 0% East and South Asian 5% South East Asian 0% Other 5% Missing 2% **Annual income** <$40,000 41% $40,000 - $80,000 26% > $80,000 26% Missing 5% **Education** < High school 7% High school graduate 24% Community college 21% University undergraduate or graduate degree 40% Other 5% Missing 2% **Employment status** Full-time 40% Unemployed 5% Part-time 17% Retired 35% Missing 2%	20% RCT
Shukla 2020 [49] Australia and New Zealand	To determine the acceptability and perceived benefit of prehabilitation in lung cancer among thoracic surgeons	Cross sectional survey	Online Prehabilitation	Thoracic Surgeons	Thoracic surgeons *n=* 28 Age 46 (SD 12.3)	40% Quantitative Descriptive
Solheim 2017 [50] Norway and United Kingdom	To assess the feasibility and potential efficacy of a multimodal intervention to attenuate cachexia in patients with incurable lung or pancreatic cancer	Randomised, feasibility trial	Home based multi modal prehabilitation	People with lung or pancreatic cancers	46 **Intervention** *n=*25 Male 60% Median age 63 [IQR 54.5–68.0] **Ethnicity** Caucasian 96% Other 4% **Control** *n=*21 Male 52.4% Median age 59 [IQR 52.5–67.0] **Ethnicity** Caucasian 100% Socioeconomic status not reported.	80% Quantitative Descriptive
Stalsberg 2022 [51] Norway	To investigate adherence to an outdoor 12-month post-surgery supervised exercise intervention during seasonal variation among newly diagnosed breast cancer patients receiving adjuvant treatment, and to identify sociodemographic and health-related adherence predictors.	Feasibility study Intervention arm of randomised trial	Outdoor exercise prehabilitation	People with breast cancer	99 **Intervention** *n=*47 54.2 [SD10.1] **Education** College/university degree > 4 years 25.5% College/university degree ≤ 4 years 29.8% High school = 3 years 27.7% Vocational training/elementary school 17.1% **Occupation** Management position public/private 14.9% Management position, academic 12.8% Lower profession 27.7% Non-professional occupation 19.1% Self-employed business/skilled, artisan 12.8% Semi-skilled, unskilled 10.6% **Household income** High 27.7% Medium 36.2% Low 36.2% **Control** *n=*53 Details not reported	80% Quantitative Non-randomised
Steffens 2021 [52] Australia	To establish the feasibility and acceptability of a preoperative exercise program, and to obtain pilot data on the likely difference in key surgical outcomes to inform the sample size calculation for a full-scale trial.	Single centre two arm randomised controlled trial	Blended exercise prehabilitation	People with gastrointestinal cancer	22 **Intervention** *n=*11 Male 54.5% Mean age 62 [48.0 to 72.0] **Control** *n=*11 Male 54.5% Mean age 66 [46.0 to 70.0] Ethnicity not reported. Socioeconomic status not reported.	100% RCT
Thoft Jensen 2019 [53] United States	To assess feasibility of an existing Danish home-based prehabilitation program when implemented in a US cancer centre	Pilot feasibility study	Home based bimodal (exercise and nutrition) prehabilitation	People with bladder cancer	32 Male 91% Age 69.3 [SD7.7] Ethnicity not reported. Socioeconomic status not reported.	80% Quantitative Descriptive
Tweed 2021 [54] Netherlands	To explore the feasibility of the BEFORE (Better Exercise and Food, Better Recovery) multimodal prehabilitation program consisting of personalized, ambulatory, hospital based exercise training, and fresh protein-rich food in terms of compliance, organization and acceptance to outline the design of a large, statistically well-powered comparative trial.	Pilot feasibility study	Hospital based Bimodal (exercise and nutrition) prehabilitation programme	People with colorectal cancer	9 Male 55.5% Mean age 73 [IQR 70.0–76.0] Ethnicity not reported. Socioeconomic status not reported.	80% Quantitative Descriptive
Van Rooijen 2019 [55] Netherlands	To test the feasibility, safety, and effectiveness of a multimodal prehabilitation program intended to be studied in a randomized controlled trial	Prospective observational cohort study	Blended, multimodal prehabilitation	People with colorectal cancer	50 **Intervention** *n=*20 Male 50% Age 75 [IQR 62–89] **Control** *n=*30 Male 57% Age 71 [IQR 46–84] Ethnicity not reported. Socioeconomic status not reported.	60% Quantitative Non-randomised
Waller 2022 [56] United Kingdom	To assess the efficacy of a tri-modal prehabilitation programme delivered by smartwatches for improving functional fitness prior to major abdominal cancer surgery.	Single centre pilot randomised controlled study	Digital multimodal prehabilitation	People undergoing major abdominal cancer surgery	22 **Prehabilitation** *n=*11 Male:Female 4:7 Age 55.5 [49.2, 61.7] **Control** *n=*11 Male:Female 7:4 Age 61 [53.1, 68.9] Ethnicity not reported. Socioeconomic status not reported.	80% RCT
Waterland 2022 [57] Australia	To investigate the feasibility of delivering a hospital- and community-based prehabilitation program in patients identified at high risk of postoperative complications	Cohort study	Hospital and community based exercise prehabilitation	People with colorectal, prostate, oesophageal, pancreatic, gastric cancers and sarcoma	50 Male 52% Median age 71 [IQR 63–77] Ethnicity not reported. Socioeconomic status not reported.	80% Quantitative Descriptive
Wu 2021 [58] United Kingdom	To determine the feasibility of multimodal prehabilitation as part of the breast cancer treatment pathway	Cohort study	Blended multimodal prehabilitation	People with breast cancer	44 **Intervention** *n=*24 Female 100% Age > 65 54% <65 46% **Ethnicity** Afro Caribbean 4% Asian 8% White British 88% **Control** *n=*20 Female 100% Age >65 40% < 65 60% **Ethnicity** White British 100% Socioeconomic status not reported.	60% Quantitative Non-randomised
Qualitative studies						
Agasi-Idenburg 2020 [59] Netherlands	To investigate the barriers, facilitators, and preferences for preoperative exercise programs in older patients scheduled for CRC surgery.	Qualitative	Cancer Institute Exercise prehabilitation	People awaiting or who had undergone colorectal cancer surgery and their informal carers Physiotherapists	33 **Patients** = *n=*11 Male 64% Mean age = 72.7 [SD 4.39] **Informal carers** *n=* 13 Male 23% Mean age 68.1 years [SD 11.98] **Physiotherapists** *n =* 9 Male 44% Mean age 42 [SD 9.96] Ethnicity not reported Socio-economic status not reported	80% Qualitative
Banerjee 2019 [60] United Kingdom	To investigate the perspectives and experiences of bladder cancer patients who participated in a programme of vigorous intensity aerobic interval exercise prior to radical cystectomy.	Qualitative	University based exercise prehabilitation	People with bladder cancer	14 Male 93% Age 72.3 [SD 6.0] Ethnicity not reported Socio-economic status not reported	100% Qualitative
Beck 2022 [61] Denmark	To investigate the experiences, thoughts, and feelings that underlie and influence actions or the lack of actions in relation to prehabilitation among cancer patients due to undergo major abdominal surgery	Qualitative Phenomenology	Home based multimodal prehabilitation leaflet	People with colorectal and ovarian cancers	16 **Colorectal** *n=*9 Male 55.5% Median age 58 **Ovarian** ***n=*7** Median age 58 Ethnicity not reported Socio-economic status not reported	100% Qualitative
Bingham 2023 [12] United Kingdom	To explore mechanisms promoting feasibility and acceptability of a MCPP from patients and professionals perspectives exploring planning, development and implementation.	Qualitative	Blended multimodal prehabilitation	People with head and neck, colorectal and lung cancers Health professionals implementing prehabilitation intervention	33 **People with cancers** ***n=*** **9** Male 67% Age group [years] 46–60 = *n =* 2 61–75 = *n =* 6 76 plus *n =* 1 Head and Neck *n =* 2 Colorectal *n=* 3 Lung *n =*4 Ethnicity not reported. Socio-economic status not reported. **Professionals (** ***n =*** **24)** Male 21% RN *n =* 7 Doctor *n =* 4 Speech and language therapist *n =* 1 Physiotherapist *n =* 2 Dietician *n =* 1 Move More Coordinators *n =* 3 Assistant psychologist *n =*1 Emotional wellbeing support *n =* 1 Health Development Manager *n =* 2 Performance Manager *n =* 1 Cancer Services Manager *n =* 1	100% Qualitative
Brady 2020 [62] United Kingdom	The aim of the project was to improve service provision of pre-treatment SLT assessment and information counselling for patients undergoing radiation treatment for HNC. The service evaluation sought to develop an improved pre-treatment assessment service designed in partnership with patients and SLTs.	Participatory action research	Hospital based SLT prehabilitation	People with head and neck cancer	14 **People with head and neck cancer** *n =*7 Demographic characteristics not reported. Ethnicity not reported Socio-economic status not reported **Healthcare professionals** *n=*7 Radiation oncologist *n=*1 Clinical Nurse Specialist *n=*2 Head and Neck Dieticians *n=*2 Speech and Language therapist *n=*2	100% Qualitative
Collaco 2021 [63] United Kingdom	To explore patients and healthcare professionals views and experiences of a pre- and post-operative rehabilitation intervention (SOLACE), for patients undergoing surgery for early-stage lung cancer.	Qualitative description	Multimodal hospital and/or community based prehabilitation	People with lung cancer	25 **People with lung cancer** *n =* 17 59% Female Age group [years] 40–49 *n =* 1 50–59 *n =* 0 60–69 *n =* 4 70–79 *n =* 9 80–89 *n =* 3 **Ethnicity** White 100% Socioeconomic status not recorded. **Healthcare professionals** *n =* 8 Nurse *n =* 4 Advanced Therapist Practitioner *n =* 1 Surgeon *n =* 1 Respiratory *n =* 1 Physician *n =* 1	80% Qualitative
Cooper 2022 [64] United Kingdom	To identify factors influencing uptake, engagement and adherence to the ChemoFit intervention and to establish whether it was acceptable and feasible to use.	Qualitative	Home based exercise prehabilitation	People with upper gastrointestinal cancers	22 82% Male Mean age 67.27 [SD 8.21] **Ethnicity** White British 100% **Index of Multiple Deprivation (deciles)** 1 *n =* 2 2 *n =* 3 3 *n =* 3 4 *n =* 1 5 *n =* 1 6 *n =* 3 7 *n =* 2 8 *n =* 1 9 *n =* 3 10 *n =* 3	100% Qualitative
Daun 2022 [65] Canada	To understand patient and HCP perspectives on the role of multiphasic exercise prehabilitation considering unique needs across the surgical timeline for HNC patients	Qualitative interview study	Hospital based exercise prehabilitation	People with head and neck cancers and healthcare professionals	20 **People with head and neck cancer** *n =* 10 Male 90% Mean age 60.8 [SD 8.5] **Ethnicity** White *n=*9 Not Specified *n=*1 **Employment Status** Disability *n =* 1 Part Time *n=* 3 Full Time *n=*4 Unemployed *n=* 2 **Annual Family Income** $60,000–79,999 *n=* 1 $80,000–99,000 *n=* 1 >100,000 *n=* 3 Prefer Not to Answer *n=* 5 **Healthcare professionals** *n =* 10 Male 40% Surgeon *n =* 4 Oncology Nurse *n =* 2 Physiotherapist *n =* 1 Unit Manager *n =* 1 Clinical Nurse Educator *n =* 1 Unit nurse/research assistant *n =* 1	100% Qualitative
Ferreira 2018 [66] Canada	To better understand patients' perspectives of prehabilitation and to identify factors related to programme adherence	Cross sectional survey	Blended multimodal prehabilitation programme	People with colorectal and lung cancer	52 Male 53.8% Mean age 66.9 [SD 12.1] Ethnicity not reported. Socio-economic status not reported.	20% Qualitative.
Hogan 2019 [67] Australia	To explore enablers and barriers for patients of overall compliance with preoperative oral nutrition supplements in patients undergoing pelvic exenteration surgery for cancer.	Qualitative	Nutritional prehabilitation	People who had had pelvic exenteration surgery for cancer	20 Male 30% Median age 62 [range 33–79] Ethnicity not reported. Socio-economic status not reported.	20% Qualitative
McCourt 2023 [68] United Kingdom	To explore the experiences of participants who took part in the PERCEPT myeloma pilot trial in order to aid the design of a fully powered RCT.	Qualitative Interview	Hospital based exercise prehabilitation	People with myeloma	16 Male 56% Mean age 61 [SD 11] Ethnicity not reported. Socio-economic status not reported.	100% Qualitative
Murdoch 2021 [69] United Kingdom	To identify recommendations for improving intervention delivery within the main trial	Qualitative (Process evaluation)	Blended exercise prehabilitation	People with colorectal cancer Healthcare professionals	41 **Patients** *n=* 29 **Hospital-Supervised Exercise** *n=* 10 Male 90% Age 65 [range 39–79] **Ethnicity** White British 90% North African 10% **Employment status** Employed 40% Retired 50% Unemployed 10% **Home-supported exercise** *n=*14 Male 58% Age 71 [range 59–85] **Ethnicity** White British 100% **Employment status** Employed 14% Self-employed 7% Retired 78% **Treatment as usual** *n=* 5 Male 80% Age 73 [68–80] **Ethnicity** White British 100% **Employment status** Employed 40% Retired 60% **Healthcare** **professionals** *n=* 13 Researchers *n=* 9 Physiotherapists *n=* 3 Exercise practitioner *n=*1	100% Qualitative
Robinson 2023 (70) United Kingdom	1. To identify frontline cancer HCP’ current views about physical activity for PABC relevant to their professional role and workplace 2. To determine if and when physical activity advice is currently part of usual practice amongst frontline cancer HCPs 3. To understand barriers and opportunities to integrating PA-based cancer rehabilitation within a range of cancer specific services, as well as eliciting HCP suggestions for future practice	Qualitative	Exercise prehabilitation	Cancer Healthcare professionals	**Healthcare professionals** *n=* 21 Nurses *n=*8 Physiotherapists *n=* 3 Haemato - Oncologists *n=* 5 Oncologists *n=* 4 GP clinical assistant *n=*1	100% Qualitative
Sun 2020 [70] United States	To determine the barriers and facilitators of adherence to a perioperative physical activity intervention in older adults with lung and gastrointestinal (GI) cancers and their family caregivers	Qualitative	Digital exercise prehabilitation	Older adults with lung and gastro intestinal cancers and their family caregivers	34 patient and family care giver dyads **Lung cancer** *n=*18 Median age 74 Focus groups median age 71 **Gastrointestinal cancer** *n=*16 Median age 68 Focus groups median age 67 Male [all patients] 59% **Ethnicity [all patients]** White 82% Other 18% Male [all carers] 41% **Ethnicity [all carers]** White 82% Other 18% **Employed [all carers**] 53%	60% Qualitative
Wu 2022 [71] United Kingdom	To describe our patients' perceptions of tele-prehabilitation and capture their capabilities, opportunities, and motivations to participate.	Qualitative descriptive	Multimodal teleprehabilitation	People with colorectal, breast and urological cancers.	22 Male 50% Age 66 [range 42–83] **Ethnicity** White 86.5% Black 9.1% Did not disclose 4.5% Socio-economic status not reported.	100% Qualitative
Mixed-methods studies						
Beck 2021 [72] Denmark	To understand perspectives on and acceptability of prehabilitation among patients undergoing complex abdominal cancer surgery	Mixed-methods	Home based multimodal prehabilitation leaflet	People with colorectal and ovarian cancers	79 **Interviews** *n =* 31 Male 39% Mean age 60 **Leaflet** *n =* 53 Male 13% Mean age 62 Five patients completed leaflets and were interviewed - thus, a total of 79 patients contributed with data (31 + 53–5 = 79). Ethnicity not reported Socio-economic status not reported.	20% Mixed-methods
Beck 2021 [73] Denmark	To investigate what patients with cancer who were due to undergo major abdominal surgery (CRS HIPEC) actually were able to do when provided with preoperative, home-based, multimodal recommendations presented in a leaflet, instead of taking part in a standardised programme.	Convergent mixed-methods	Home based multimodal prehabilitation leaflet	People with colorectal and ovarian cancers	53 **Colorectal** ***n =*** **20** 56% Female Mean age 66 [SD 8.53] **Ovarian** ***n =*** **33** 100% Female Mean age 59.5 [SD 12.42] Ethnicity not reported Socio-economic status not reported.	40% Mixed-methods
Brahmbhatt 2020 [74] Canada	To assess the feasibility and acceptability of an individualized, home-based prehabilitation intervention prior to breast cancer surgery.	Emergent mixed methods	Home based exercise prehabilitation	People with breast cancer Healthcare professionals	**Patients** *n =* 22 Mean age 54.18 [SD 10.98] **Ethnicity** White/Caucasian *n =* 14 Latino/Hispanic *n =* 2 East Asian *n =* 2 South East Asian *n =*1 South Asian *n =* 1 Ashkenazi Jewish *n =*1 Prefer not to answer *n =*1 **Education** Finished University/college *n =*15 Some University/college *n =*3 Some high school *n =* 1 Other *n =* 2 Prefer not to answer *n =*1 **Working status** Working/studying full-time *n =*11 Working/studying part-time *n =*2 Retired *n =* 2 Unemployed *n =* 1 Disability/sick leave *n =* 2 Other *n =*3 Prefer not to answer *n =*1 **Socioeconomic status** >$75,000 *n =* 9 $40,000–$75,000 *n =*2 $20,000–$39,000 *n =* 2 <$20,000 *n =* 3 Prefer not to answer *n =*6 **Health professionals working in breast cancer clinic** *n=*2	40% Mixed-methods
Deftereos 2022 [75] Australia	To analyse the implementation of a standardised nutrition care pathway for UGI cancer surgery into clinical practice from the perspectives of dietitians, multi-disciplinary team (MDT) members and patients using a validated theoretical framework, the Consolidated Framework for Implementation Research (CFIR)	Mixed methods Convergent parallel	Hospital based nutritional care prehabilitation	People with upper gastro intestinal tract cancers Healthcare professionals Dieticians	**Satisfaction survey** Patients *n=*18 Healthcare professionals *n=*14 **Focus groups** Dieticians - exact number of participants not known. Ethnicity not reported. Socio-economic status not reported.	40% Mixed-methods
Low 2020 [76] United States	To develop and test a mobile technology-supported intervention to reduce sedentary behaviour before and after cancer surgery, and to evaluate the usability and feasibility of the intervention.	Mixed-methods	Digital exercise prehabilitation	People scheduled for surgery for metastatic colorectal or peritoneal cancer	15 Male 20% Age 49.7 [range 25–65] **Ethnicity** White: 87% Black: 13% **Employment status** Full time work 40% Part time work 13% Retired/not working 47% **Education** High school diploma or equivalent 27% Some college 33% Bachelor’s degree or higher 40%	20% Mixed - methods
Macleoud 2018 [77] United Kingdom	To assess the practical aspects of delivering and evaluating a lifestyle intervention programme (TreatWELL) for patients with CRC undergoing potentially curative treatment.	Mixed-methods	Hospital based multimodal prehabilitation	People with colorectal cancer	22 Male 77% Age 67.0 [IQR 60.0- 74.3] **SIMD** 1–3 [most deprived] 23% 4–7 45% 8–10 [most affluent] 32% Ethnicity not reported.	20% Mixed- methods
Mawson 2021 [78] United Kingdom	To assess the acceptability of the intervention through qualitative interviews and retention rates during the study.	Mixed-methods	Hospital based exercise prehabilitation	People with myeloma	13 Male 70% Mean age 65 [range 53–78] Ethnicity not reported. Socio economic status not reported.	20% Mixed - methods
Provan 2022 [79] United Kingdom	To document current practice, barriers and challenges to implementing prehabilitation in order to provide insight for the development of national frameworks for action and co-ordinated evaluation procedures in Scotland.	Mixed-methods	Prehabilitation interventions	Cancer care stakeholders	**Surveys** *n=*295 NHS Employees 95% Medical staff 35% AHPs 33% Nurses 15% **Healthcare Professional Interviews** *n=*11 Detail on demographic characteristics not reported.	20% Mixed - methods
Waterland 2021 [80] Australia	To evaluate the current and likely future impact of a telehealth preoperative education package for patients preparing for major abdominal cancer surgery using the RE-AIM framework with the exception of the maintenance dimension	Mixed-methods	Digital education prehabilitation	Adults scheduled to have major abdominal cancer surgery	35 Male 46% Mean age 59 [SD 9] **Education** Primary school 6% Secondary school 34% Trade school/TAFE 25% Undergraduate degree 22% Postgraduate degree 13% Ethnicity not reported.	40% Mixed-methods

### Study characteristics

Of the 32 quantitative studies reviewed, there were eight randomised controlled trials, two single-arm multi-centre trials, seven cohort studies and one cross-sectional survey. Others were pilot (*n =* 3), feasibility (*n* = 7), observational (*n* = 1) and prevalence (*n* = 1) studies, with one non-randomised trial and one audit. Qualitative studies (*n* = 15) mainly used a broad qualitative approach (*n* = 12), one used phenomenology, one participatory action research and one used a cross-sectional survey. Nine studies used mixed methods.

### Study populations

The majority of included studies were conducted in Europe (*n* = 33) (UK (*n* = 19), Netherlands (*n* = 4), Denmark (*n* = 3), Spain (*n* = 1), France (*n* =1), Portugal (*n* = 1), Belgium (*n* = 1), Slovenia (*n* = 1), Norway (*n* = 1) and Sweden (*n* = 1)). Eleven were conducted in North America (Canada (*n* = 8), United States (*n* =3)), and eight were from Australia. The remaining studies were from Japan (*n* = 1) and China (*n* =1), and two studies were conducted across two countries, Australia and New Zealand and the UK and Norway. Studies focused on prehabilitation in different settings including hospitals (*n* = 12), local communities (including universities and local gymnasiums), individuals’ homes (*n* = 14) and outdoors (*n* = 1). Ten studies reported a hybrid, home and hospital approach to prehabilitation, whilst digital prehabilitation was reported in nine studies. Fifty-three studies were conducted in a range of cancers. Of these, 41 reported data for a single cancer site: colorectal (*n* = 11); gastrointestinal (*n* = 9); lung (*n* = 7); haematology (*n=*4); breast (*n* = 3); head and neck (*n* =2); bladder (*n* = 2) prostate (*n*=1) and a range of abdominal surgeries (*n* = 3). In 12 studies, cancer sites were pooled. Three studies focused on healthcare professionals (*n* = 2) and key stakeholders (*n* = 1).

### Methodological quality

There was considerable variation in the methodological quality of the 56 studies included. Twelve studies, 10 qualitative and two quantitative, satisfied all the MMAT criteria [23]. Fourteen studies, nine mixed methods, two qualitative and three quantitative, satisfied just one or two criteria. Thus, data were extracted from a body of literature where one-fifth (21%) of publications were about research of the highest quality, defined as having met 100% of the MMAT criteria [23]. Detailed results of the MMAT quality assessments are found in supplementary information (supplementary information 3).

### Thematic synthesis

**Fig. 2:**
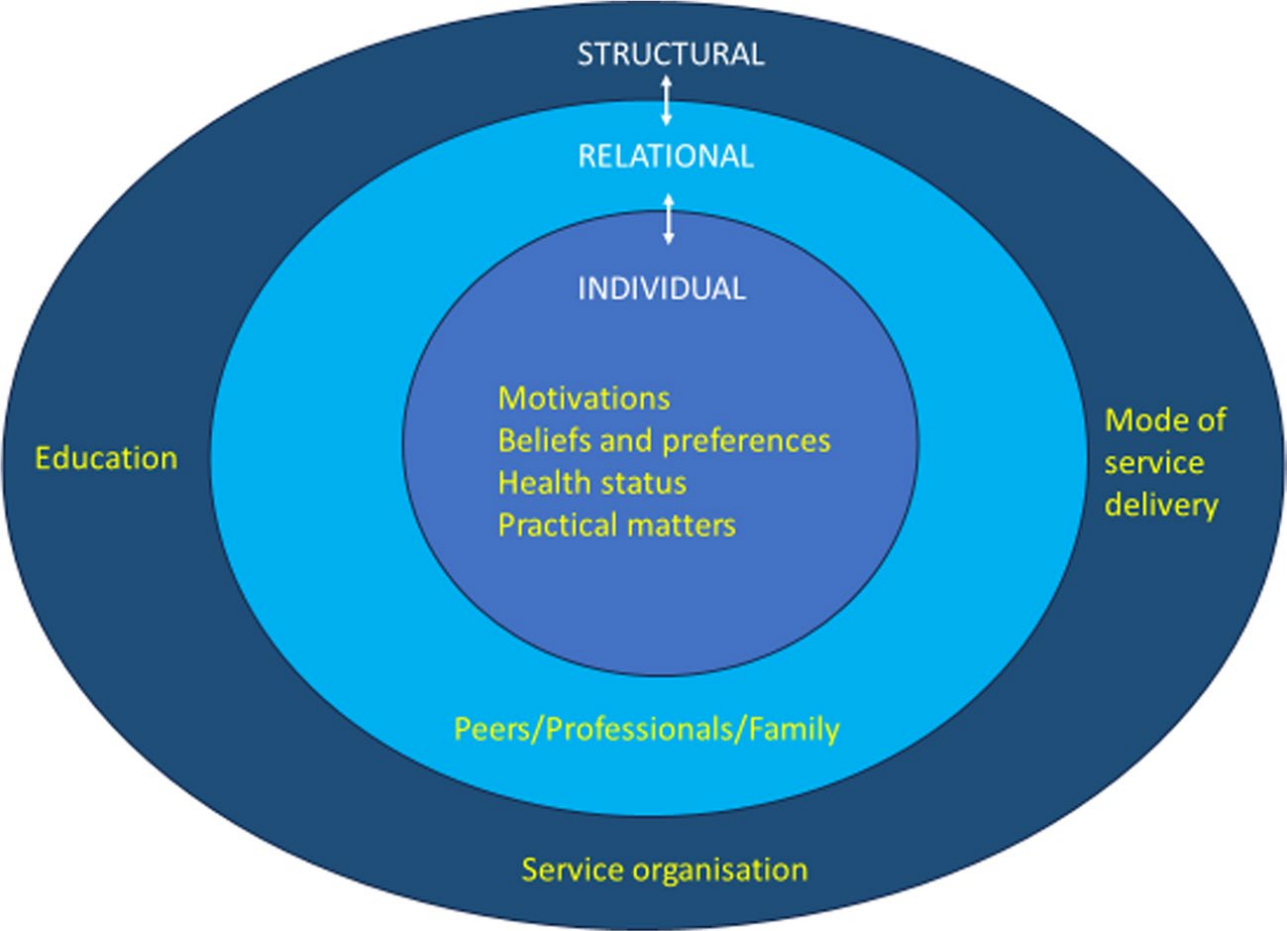
Overarching themes

The thematic synthesis identified three cross-cutting analytic themes. As illustrated in Figure 2, these themes reflected individual, structural and interpersonal facilitators of and barriers to access, acceptability and adherence of cancer prehabilitation:

Theme 1 *The influence of individual drivers of cancer prehabilitation engagement*

Theme 2* Providing acceptable cancer prehabilitation service and interventions*

Theme 3* Interpersonal support – the unifying golden thread*

Interpersonal support was the unifying golden thread as it facilitated the fit between the individual and the structural for access to, acceptance of and adherence to prehabilitation.

#### Theme 1. The influence of individual drivers of cancer prehabilitation engagement

Factors at the level of the individual were found to shape prehabilitation access, acceptance and adherence. These included perceived need and benefits, motivations, health status and everyday practicalities.

##### The perceived need for and potential benefits of prehabilitation

A key stimulus for accessing and adhering to cancer prehabilitation was a belief that engagement might confer benefit. Influences included clinicians’ prehabilitation endorsement and encouragement [12, 13, 42, 52, 55, 59, 60, 65, 66, 71], positive prior personal experiences of routine physical activities [60, 69, 70, 77] and weight loss programmes [77], other patients’ support [12, 71] and the perceived need to improve personal fitness [60, 63]. Some participants in UK-based studies believed they had a social responsibility to engage in prehabilitation [63, 64] as enhanced fitness would benefit healthcare services financially [12, 64].

The money, the cost per night in the hospital, goodness knows how much that costs and the follow-up with all the doctors, the dieticians and everyone else behind (….). It’s (prehabilitation) saving the NHS thousands and thousands of pounds of money ([64] p.4).

Several studies indicated some individuals perceived prehabilitation to be beneficial in that interventions provided a welcome distraction from their illness and situation [64, 72, 74]. Benefit was understood in terms of being psychologically and physically prepared for cancer treatments, potentially enhancing post-treatment recovery and survival [12, 55, 60, 63, 64, 66–68, 70, 71, 74].

I benefited a lot from it because it caught me in that time just after diagnosis when things were pretty scary and pretty awful and I felt like it was one of the key pieces of my plan for positivity during this whole thing, because it was setting a tone for recovery ([74] p. 8)

Yet, it was also clear that some individuals were disinterested in engaging with prehabilitation [56, 58, 66, 74, 80]. Some studies suggested a connection between imminent surgery and patients’ perceptions of little benefit of prehabilitation in the short timescales [47, 54, 63, 69, 77, 79]. Some individuals felt that making additional hospital visits for prehabilitation was onerous [54]. Others were unaccustomed to or did not want to exercise [36, 70] or perceived exercise as demanding [41], particularly when combined with cancer treatment [51]. Some considered their existing fitness levels [61, 63] and diet [61] sufficient. A sense of low perceived benefit of or need for prehabilitation meant it was considered a low priority [36].

##### Personal motivators

A cancer diagnosis [71, 77] conjoined with the desire to improve fitness [63, 64, 72], survive surgery [63, 64] and to be present for and enjoy their families [64] were influential motivators for individuals’ proactively effecting lifestyle change and thus engagement with prehabilitation. Having accessed prehabilitation, exercise logs and diaries [64, 68, 74], personal goal setting [61, 64, 71], progress self-monitoring [61, 64, 68, 71, 77], activity tracking and objective feedback [56, 60] motivated individuals to maintain participation. They inspired them to remain on track, enabled them to realise their progress, build self-efficacy for prehabilitation adherence [60, 70, 73, 76, 77] and, through a process of cognitive reframing, regain a sense of control [71].

Now I have a feeling of control over my body . . . I don’t want cancer to define me. [71]

Nonetheless, one study reported that motivation to access prehabilitation may be negatively affected by low levels of health literacy, which is associated with socioeconomic deprivation [46]. Furthermore, sustaining motivation to continue prehabilitation could be challenging [43, 45, 58, 64, 70, 74], especially when faced with unanticipated setbacks such as delayed surgery [57] or insufficient peer support [64].

##### The enduring problems of health limitations

Individuals’ physical and psychological health status influenced prehabilitation access and adherence, particularly when there was a perception of insufficient on-going professional [61, 72, 73] and family support [31], and interventions were located away from home. Pancreatic cancer [33] adversely affected individuals’ access to prehabilitation. Furthermore, physical health problems limited some individuals’ ability to travel and thus access hospital-based prehabilitation [54, 59, 71]. Symptoms experienced and perceived health status influenced individuals’ prehabilitation adherence. Reported adherence barriers included physical symptoms [61, 67, 70, 72, 73, 81] such as fatigue [45, 50, 57, 70, 73], pain [40, 45, 57, 59, 70, 71, 73], digestive problems [30, 35, 39, 47, 55, 67] and feeling unwell [40, 43, 64, 79]. In addition, functional limitations [63, 70] associated with comorbidities [31, 37, 40, 49, 51, 57, 64, 70, 77], disease status [37, 41], pre-surgery neoadjuvant treatments [37, 53, 64, 70, 81] and mental health problems [35, 39] were all reported to negatively affect individuals’ ability to engage with and adhere to prehabilitation, particularly in terms of physical activities.

Several studies reported that psychological distress had a negative effect on prehabilitation access and adherence [59, 61, 70, 73]. Described by a participant in one study [63] as ‘dark moments’, as anxiety and stress were often connected with attending hospitals [71]. In addition, several studies reported that individuals felt overwhelmed, both generally [42, 57, 74] and emotionally [12, 70], in advance of their treatments. Information overload [62] and competing personal matters which required their attention pre-treatment [70, 80] contributed to the sense of feeling overwhelmed.

##### The challenges of everyday life

Across studies, insufficient time for prehabilitation was frequently reported [40, 50, 51, 55, 58, 66, 71, 72, 74, 77, 78]. Some individuals described competing priorities in the short space of time between diagnosis and treatment [49, 57, 59, 70, 79]. This was partly due to putting affairs in order, prioritising family time [61] or treatments being scheduled earlier than originally planned [35, 54, 55]. Others were constrained by their employment [51, 70, 73, 80] and family responsibilities, including caring for other family members [55, 58, 70]. Additional barriers to prehabilitation engagement included geographical distance to hospitals delivering prehabilitation [28, 32, 41, 51, 54, 57, 63, 74]; transport difficulties [29, 49, 51, 54, 58, 60, 66, 79] and associated financial costs [51, 66, 71]; inclement weather, particularly in relation to prehabilitation with outdoor exercise components [45, 57, 64, 70, 73, 74]; low digital literacy [34, 42, 76]; restricted or limited access to and problems with technology [42, 56, 76, 80], notably broadband [45, 79] and experiencing physical discomfort with exercise equipment [60, 64].

#### Theme 2. Providing acceptable cancer prehabilitation service and interventions

The prehabilitation environment, mode of delivery (which might be technological) and the perceived utility of interventions were important facilitators of access [34, 48, 57, 66, 71, 75, 80] and adherence [36, 45, 48, 61] and influenced acceptance [36, 52, 61, 64, 69, 71, 77, 80, 81].

##### The value of home-based prehabilitation

Home-based prehabilitation interventions with remote professional supervision and support were accepted for their convenience [38, 74], capacity to motivate [38, 61, 64, 73] and build self-efficacy [40, 61, 64, 73] and perceived benefit [40, 69, 74]. Specifically, individuals reported that home-based prehabilitation enabled them to integrate interventions into their everyday lives [61, 64]. Exercising in the safe, private, space of home was enjoyable [36, 66], could help with overcoming self-consciousness and engendered a sense of control [61, 64].

I couldn’t go to the gym any longer. I can’t very well be running out to the toilet the whole time. So, I had to find something else, so it was that [static bike at home]. ([61] p. 206)

…I don’t want to do it [prehabilitation] in a hospital because I think it then becomes really competitive. And people are, like, if they can’t do it, they feel…. They would feel like, ‘Oh, I’m not strong enough…’ you know what I mean. It might depress them. Whereas if you do it in the house, you can do it at your own pace, there’s nobody watching over you and everything. [64]

Home-based prehabilitation interventions were important facilitators of access [48, 66] and adherence [36, 48, 61]. The provision of portable exercise equipment such as resistance bands enabled sustained adherence, particularly when individuals were temporarily away from home [74]. Some individuals welcomed the freedom and flexibility of home-based prehabilitation [72]. Yet despite being provided with resources to monitor [34, 42, 52, 64, 66, 76], supplement and continue physical activity at home [48, 63, 66, 74, 77], insufficient in-person healthcare professional engagement and encouragement could mean adherence was often difficult to monitor [69, 81] and sustained intervention adherence could be challenging [28, 63, 64] and afforded a low priority by individuals [61, 72, 73].

There had to be real pressure, there really had! And then if suddenly they were not around (the health professionals), then I’m not sure I’d finish it. That’s how I am. You have to keep an eye on me. [72]

##### Navigating the technological space of tele-prehabilitation

Sometimes referred to as ‘tele’ or ‘digital’-prehabilitation, technology-based uni and multimodal home-based prehabilitation capitalised on internet and/or telephone communication services and was delivered using smartphones, videos, wearable technology, tablets, mobile applications, video platforms and secure video conferencing [34, 36, 42, 45, 56, 70, 71, 76, 80]. In terms of acceptability, individuals perceived home-based, tele-prehabilitation programmes as accessible, particularly during the SARS-CoV-2 pandemic [34, 71, 80]:

Having prehabilitation outside of the hospital setting made things easier. I wasn’t feeling good with the pain and couldn’t travel too far. Could also do it in my own time ([71] p. 646)

Home-based tele-rehabilitation was also perceived as motivating [36, 45, 56, 76], conferred benefit [34, 36, 45, 56, 80], particularly when personalised [34, 45, 56, 71] and reduced transport-associated costs [80].

Sustained tele-prehabilitation engagement was aided by the provision of smartphones [56, 76], tablets with relevant applications and content downloaded [34], training watches [34, 56, 76], supplementary information and alternate web browser pathways for those without access to or with low digital literacy [42] and integrated digital training and support during the intervention’s implementation [34, 36, 42].

I would not have been able to endure the treatments and the surgery thereafter had it not been for the continuous support I was receiving through the digital platform. [34]

Reported barriers were primarily intervention specific. They included technical [45, 80] and device connectivity issues [34, 76], broadband and website interface problems, particularly for individuals unaccustomed to using technology [45]. Negative views of mobile mindfulness apps [56] and equipment aesthetics [76] were also described.

##### The perceived utility of prehabilitation interventions

Interventions that were perceived as being accessible in terms of their user-friendliness [34, 56, 74, 76] and appropriately designed to meet individuals’ needs, preferences and capabilities in terms of their structure [40, 52, 60, 68, 74, 77, 78], notably coherence [36, 38, 45, 75, 76] and components [38, 54, 55, 64, 69, 74], including nutritional supplements [44, 54, 55, 67], enhanced acceptability. The acceptability of prehabilitation interventions was reflected in the expressions of gratitude [12] and the positive ways in which interventions were variously described by individuals in some studies [12, 38, 58, 64, 74] as ‘excellent’, ‘very good’, ‘great’, ‘brilliant’, ‘hugely beneficial’ and ‘fun’. Some would even recommend home-based prehabilitation to people preparing for cancer treatments [52, 63, 68, 74]. However, one study [42] reported that unfamiliarity with the English language had a negative impact on access, whilst in another study [56], individuals reported adhering to protein targets challenging.

At an individual level, the availability [61] and extent of integrated healthcare professional supervision and support was perceived to enable intervention access [75] and adherence [42, 60, 61, 64, 66, 68, 69, 74, 78], particularly when this was personalised [34, 45, 56, 65, 68, 71, 78]. Unpalatable nutritional interventions had a negative effect on intervention adherence [30, 50], and it was reported that inspiratory muscle training devices could be difficult for individuals to use [38].

Healthcare professionals reported organisational barriers to implementation, and thus individuals’ access to, acceptance of and adherence with prehabilitation. These barriers included workforce capacity limitations [12, 65, 75, 79, 81], including insufficient embedded specialist prehabilitation professionals [69, 81], delayed or insufficient referral to prehabilitation [33, 44, 63], disconnect in cross-boundary systematic service delivery and communication [12, 28, 75, 81], inadequate funding [12, 65, 79, 81] and awareness of local prehabilitation provision, uncertainty regarding what constitutes prehabilitation among some healthcare professionals [28, 79, 81] and space and time constraints [69, 81] together with insufficient equipment [28] in hospital settings to deliver interventions [81].

#### Theme 3. Interpersonal support: the unifying golden thread

Across the studies reviewed, the unifying golden thread was interpersonal support, for this was an important, valued enabler of prehabilitation access [64] acceptance and adherence. It was reported that interpersonal support was derived from family and friends [12, 45, 60, 61, 64, 70, 73], prehabilitation healthcare professionals [42, 51, 55, 60, 61, 63, 64, 66, 69, 71, 75, 78], prehabilitation peers [51, 59], volunteers [79] and in-person and online peer support groups [71, 79]. When embedded within interventions, a network of interpersonal support helped to sustain prehabilitation adherence, particularly in relation to physical activity [59, 60, 68, 72, 79]. During what could be challenging times, the interpersonal support experienced during prehabilitation enhanced interventions’ acceptability [52, 60, 63, 68].

The active involvement of family during physical activities such as walking and exercise routines was reported to generate a sense of companionship, encouragement and motivational and psychological support [34, 60, 61, 64, 70, 71, 77]. In these ways, prehabilitation interventions with embedded family support enhanced their acceptability [52].

My wife did the same ones with me so there were two of us doing the same stuff. We did the walks together. Then we would both do the exercises. So that was good company. [64]

Findings reported in one study [31] indicated that living alone could have a negative effect on prehabilitation adherence.

The acceptability of prehabilitation interventions was enhanced by relevant healthcare professionals’ supportive dialogue in the shape of information, personalised encouragement, validation and timely, constructive feedback on individuals’ engagement, progress and performance [69, 77], signposting to other support services [63] and broader emotional support [77]. In addition to sustaining prehabilitation behaviours through collaboration, activation and motivational support [60, 61, 71, 72, 77, 78], healthcare professionals’ presence instilled a sense of trust [71], comfort [51] and safety [38, 62, 63] and reduced feelings of social isolation [71]. The need for and importance of supportive dialogue with healthcare professionals during prehabilitation was identified by participants in one study investigating individuals’ experiences of multimodal prehabilitation delivered via a leaflet and with no embedded healthcare professional support [73].

I have only been a number. Like I was a garden shovel with a barcode that you scanned at the cash register. There is no one who thinks about what this means for one’s self-understanding–- just to be regarded as a disease [...] There is no one asking about the human being behind it. It is insane [73]

For some participants, peer support in the shape of information sharing was beneficial and enabled prehabilitation access [63, 71]. Integrated group or one to one peer support was reported to enhance an intervention’s acceptability [12, 63]. In part, this was because individuals did not always want to engage their families, and peer support reduced their sense of isolation [71]. Peer support was reported to be beneficial in terms of interaction with others in a similar situation, thereby lending individuals’ social, emotional and motivational support, enabling them to remain on track with their prehabilitation programme [51, 59, 64, 66, 71].

Exercising in a group motivates. Let new patients exercise with other patients who are further along and have more experience exercising. They (experienced patients) can then tell them, Yes, you will get muscle aches, but they will subside too. [59]

It was clear from some studies that the absence of peer support in prehabilitation interventions was lamented [64, 71], with some participants exercising agency and accessing online patient forums to derive required support [71].

## Discussion

This review reports findings from across the globe regarding facilitators of and barriers to access, acceptance and adherence of cancer prehabilitation. The findings draw attention to cross-cutting themes at individual and structural levels and interpersonal factors that connect the levels. As illuminated in Fig. 2, the multifaceted facilitators and barriers underscore the complexity of cancer prehabilitation access, acceptance and adherence.

This review found interpersonal connections, support either directly obtained from peers, family, healthcare professionals or via digital connectivity, can facilitate a fit between the individual factors and structural factors that affect engagement with prehabilitation. Examples include encouragement from a spouse willing to engage in a recommended physical activity with the patient, practical help with digital technology, peer support during group prehabilitation and health professional supervision. Support through these interpersonal connections may be a core ingredient for successful access, acceptance and adherence. This proposition should now be explored and tested. There may be sub-groups with need or preference for certain sources of interpersonal support. Our review was designed to find out ‘what is known about access, acceptance and adherence to cancer prehabilitation, particularly among socially deprived and minority ethnic groups’ because of the known benefits from prehab for post treatment recovery [8, 9]. It found no empirically based analysis of prehabilitation access, acceptance or adherence by people from these groups.

### The individual and structural context

This review revealed individual factors enabling or impeding prehabilitation access, acceptance and adherence include personal beliefs and understandings about potential harms or benefits; motivations, for example finding enjoyment in participation; health status and everyday practicalities such as time and transport availability. Structural factors identified included the availability of knowledgeable and supportive health professionals and/or people affected by cancer’ service organisation, such as the availability of a prehabilitation multidisciplinary team and the place and space of service delivery, for example, if it was available in the community.

Individual and structural level factors affecting access to cancer treatment and care are widely reported [82–85]. Some are proposed to be modifiable for improved health outcomes in groups at risk of poor health because of poverty and/or discrimination based on age, race, ethnicity or gender [84]. The findings of the review are consistent with this wider literature on service access, acceptance and adherence. It is notable that although our search was designed to identify all literature about access, acceptance and adherence to cancer prehabilitation from 2017 to 2023, we found no analysis of structural differences. The differential experience of people from structurally vulnerable groups, for example, those who are socioeconomically deprived or from minority communities, had not been considered. Yet, evidence indicates that cancer rehabilitation services are underutilised by people from socioeconomically deprived communities [86, 87] and ethnic minorities [88]. We also know patient engagement with prehabilitation is variable [89], and third sector organisations claim people from socioeconomically deprived communities, which include people from some ethnic minorities, are underserved by prehabilitation services [1]. Exploration and understanding of difference in prehabilitation experiences across social groups is needed if support for access, acceptance and adherence is to achieve equity in health outcomes.

### Interpersonal connections linking individual experience and structural context

This review identified that it was people, namely peers, family members and friends, who, through their support, influenced the extent to which individual and structural level factors were obstacles or enablers of prehabilitation. In the relational space between individual experience and the infrastructure in place to enable prehabilitation, these people were supportive actors, influencing individuals’ access to, acceptance of and adherence to prehabilitation. 

International studies have revealed that interpersonal support is related to mental and physical health. Low perceived social support has been shown to be associated with mental and physical health problems [90]. In the USA, a high level of perceived social support was found more likely in women and young people and low level of perceived social support more likely for those living in poverty [90]. Loneliness has been proposed the mediating factor between socioeconomic status and health in a Norwegian population-based study of people aged over 40 years [91]. Two explanations were suggested. Firstly, people with few social contacts have low levels of physical activity. Secondly, people with poor physical or emotional health are more likely to have low self-esteem and self-efficacy in self-care, which is associated with less successful occupational career and low socioeconomic status and thus fewer social contact resources to manage health [91].

This review supports an argument that interpersonal connections can be important for prehabilitation access, acceptance and adherence. It found evidence of relationships with family, peers and cancer care staff influencing access to, acceptance of, and adherence to prehabilitation. Perceived social support may have a key role in successful prehabilitation. This proposition should be further explored, paying attention to the known relationship between social support and socioeconomic status in other contexts and the potential for this to be an explanation of any observed difference in access across socioeconomic groups.

### Technology as interpersonal connection?

An interesting finding is of data showing some people find web-based resources and/or online help to satisfy their prehabilitation information and support needs. These people experienced interpersonal connection through technology. An online survey among 1037 adults (18+) in the UK found that 80% of those with a long-term condition used technology for managing their health, a majority for seeking information whilst a third used wearable technology or apps. Those most likely to use technologies were younger and/or of high socioeconomic status, leading the authors to caution completely digital approaches because of the potential to exclude some groups from the care they need [92]. Arguably, technology may provide a partial solution to enabling successful prehabilitation.

### What this review adds

Our finding of structural and individual level factors affecting access to, acceptance of and adherence to prehabilitation is consistent with Levesque et al.’s [93] socioecological model of access to health services. Levesque et al.’s [93] model sets out access as a process with five dimensions of accessibility (approachability; acceptability; availability and accommodation; affordability; appropriateness) and five corresponding abilities of populations (ability to perceive; ability to seek; ability to reach; ability to pay; ability to engage). The model enables attention to social, service organisation and person-centred factors that influence access. However, the model does not address the relational dimensions derived from our data analysis, i.e. how person-centred and structural factors interrelate for better or poorer service access. Based on our findings, an important ingredient for improving access to prehabilitation may be attention to what happens in the relational space connecting these factors. Voorhees et al. [94] interpreted findings of participatory research about access to general practice and claimed it is the human abilities of workforce and clients that are an important yet absent consideration in Levesque’s model. They argued that staff training and support for human interaction were needed. We agree. In addition, and based on our analysis, we also consider important the network of interactions between patient and others. Understanding the nature and mechanisms of these interactions may be important for health equity in prehabilitation.

## Strengths and limitations

A strength of this review is that established, rigorous systematic review processes were followed to identify and select relevant peer-reviewed literature. Methods and thematic synthesis procedures were reported explicitly, providing an audit trail for dependability. To maximise study identification, the detailed and comprehensive search strategy was developed with the assistance of an expert information specialist, and the review was conducted by a multidisciplinary team with a minimum of two reviewers engaged in the screening and extracting process. Searches were limited from 2017 to 2023 and published in the English language. By limiting the search dates in this way, we have ensured that the evidence assessed has context and relevance to current policy and practices. This systematic review, as a result, provides an overarching picture and holistic understanding of access, acceptance and adherence to cancer prehabilitation. However, this review is not without its limitations. It is possible that some potentially useful studies, notably those not published in the English language have been omitted. Furthermore, we did not take account of study quality in our analysis. To reduce the risk of selection bias, studies were included irrespective of their methodological quality assessment. However, this means that some low quality evidence has been included, and this is a limitation to the credibility of the analysis. Nevertheless, there is some consistency between studies and across international healthcare settings. This does indicate a level of trustworthiness in the review findings. The review was of mixed cancer sites. Cancer site along with its symptoms and treatment-related problems may affect access, acceptance and adherence to prehabilitation. As the body of literature about engagement with prehabilitation grows, further work will be warranted to investigate cancer site–specific factors affecting inclusion in prehabilitation.

## Conclusion

ThQueryere is limited empirical study of barriers and facilitators to inform improvement in equity of access to cancer prehabilitation. To enhance the inclusivity of cancer prehabilitation, adjustments may be needed to accommodate individual preferences and characteristics, such as comorbidity, and attention given to structural factors, such as staff training. Based on our findings, we propose interpersonal connections as a fundamental core ingredient for facilitation of prehabilitation access, acceptance and adherence.

## Systematic review registration

This systematic review was registered in PROSPERO (CRD42023403776)

## Supplementary Information

Below is the link to the electronic supplementary material.

Supplementary file1 (DOCX 59 KB)

Supplementary file2 (DOCX 99.3 KB)

Supplementary file3 (DOCX 42.6 KB)

## Data Availability

All data generated for this review are included in the manuscript and/or the supplementary files.
