# Supplementary material for: Access, acceptance and adherence to cancer prehabilitation: a mixed-methods systematic review
Source: J Cancer Surviv. 2024 May 6;19(6):1895–923. doi: 10.1007/s11764-024-01605-3 (PMC12546383; doi:10.1007/s11764-024-01605-3)
Supplement: Supplementary file 1 — Supplementary file1 (DOCX 59 KB) [file 11764_2024_1605_MOESM1_ESM.docx]

**Medline (Ovid) 31^st^ May 2023**

| **Search Number** | **Search** | **Results** |
| --- | --- | --- |
| 1 | exp Neoplasms/ | 3,836,097 |
| 2 | (cancer* or malignan* or carcino* or adenocarcinoma* or neoplas* or tumo?r* or oncolog*).tw. | 4,023,272 |
| 3 | exp Radiotherapy/ | 206,852 |
| 4 | exp Chemoradiotherapy/ | 19,787 |
| 5 | exp Antineoplastic Agents/ | 1,241,599 |
| 6 | exp Chemotherapy, Adjuvant/ | 46,215 |
| 7 | exp Immunotherapy, Adoptive/ | 13,461 |
| 8 | (chemotherap* or chemorad* or chemo-rad*).tw. | 510,371 |
| 9 | ((radiation or radio* or irradiat*) adj2 (therap* or treatment*)).tw. | 142,976 |
| 10 | (check point inhibitor* or checkpoint inhibitor* or check point blockade or checkpoint blockade).tw. | 30,619 |
| 11 | OR 1-10 | 5,737,458 |
| 12 | exp Preoperative Exercise/ | 403 |
| 13 | prehab*.tw. | 1,305 |
| 14 | 12 OR 13 | 1,357 |
| 15 | (pre-operative or preoperative or peri-operative or perioperative or pre-surg* or presurg* or pre-treatment or pretreatment).tw. | 679,938 |
| 16 | exp Preoperative Care/ | 72,803 |
| 17 | exp Perioperative Care/ | 158,528 |
| 18 | exp Perioperative Period/ | 102,411 |
| 19 | OR 15-18 | 848,006 |
| 20 | exp Exercise/ | 245,091 |
| 21 | exp Exercise Therapy/ | 62,945 |
| 22 | exp Physical Therapy Modalities/ | 177,743 |
| 23 | exp Diet/ | 327,440 |
| 24 | exp Nutrition Therapy/ | 113,402 |
| 25 | exp Psychotherapy/ | 217,817 |
| 26 | exp Mind-Body Therapies/ | 47,110 |
| 27 | exp Adaptation, Psychological/ | 139,427 |
| 28 | (exercise* or physical activit* or rehabilitation or training or nutrition* or malnutrition or diet* or optimi#ation).tw. | 2,117,773 |
| 29 | ((psych* or emotion*) adj2 (support* or therap* or intervention* or education* or well-being or wellbeing or resilience or adjustment*)).tw. | 102,110 |
| 30 | OR 20-29 | 2,757,329 |
| 31 | 19 AND 30 | 67,403 |
| 32 | ((multimodal or multi-modal or multidimensional or multi-dimensional or multidisciplinary or multi-disciplinary or multicomponent or multi-component) adj (rehab* or support* or therap* or intervention* or manage*)).tw. | 16,557 |
| 33 | 14 OR 31 OR 32 | 84,116 |
| 34 | exp "Treatment Adherence and Compliance"/ | 274,874 |
| 35 | exp Health Services Accessibility/ | 134,362 |
| 36 | (adherence or engage* or accept* or motivat* or support* or participation* or feasib* or action* or access* or uptake* or approachability or availab* or compliance or barrier* or attendance or retention or completion or attrition or involvement or awareness).tw. | 7,048,608 |
| 37 | (patient* adj3 (preference* or value* or involvement* or perspective* or assessment*)).tw. | 168,669 |
| 38 | OR 34-37 | 7,318,036 |
| 39 | 11 AND 33 AND 38 | 8,449 |
| 40 | limit 39 to yr="2017 -Current" English Language | **4,248** |

**EMBASE (Ovid) 2^nd^ June 2023**

| **Search Number** | **Search** | **Results** |
| --- | --- | --- |
| 1 | exp Neoplasm/ | 5,981,900 |
| 2 | (cancer* or malignan* or carcino* or adenocarcinoma* or neoplas* or tumo?r* or oncolog*).tw. | 5,793,232 |
| 3 | exp Radiotherapy/ | 724,613 |
| 4 | exp Chemoradiotherapy/ | 75,693 |
| 5 | exp Antineoplastic Agent/ | 2,870,560 |
| 6 | exp Chemotherapy/ | 845,432 |
| 7 | exp Cancer immunotherapy/ | 107,385 |
| 8 | (chemotherap* or chemorad* or chemo-rad*).tw. | 838,959 |
| 9 | ((radiation or radio* or irradiat*) adj2 (therap* or treatment*)).tw. | 239,609 |
| 10 | (check point inhibitor* or checkpoint inhibitor* or check point blockade or checkpoint blockade).tw. | 56,018 |
| 11 | OR 1-10 | 8,780,990 |
| 12 | exp Preoperative Exercise/ | 1,181 |
| 13 | prehab*.tw. | 2,165 |
| 14 | 12 OR 13 | 2,554 |
| 15 | (pre-operative or preoperative or peri-operative or perioperative or pre-surg* or presurg* or pre-treatment or pretreatment).tw. | 977,797 |
| 16 | exp Preoperative Care/ | 51,841 |
| 17 | exp Perioperative Care/ | 167,080 |
| 18 | exp Preoperative Period/ | 423.243 |
| 19 | exp Perioperative Period/ | 1,189,796 |
| 20 | OR 15-19 | 1,832,850 |
| 21 | exp Exercise/ | 456,484 |
| 22 | exp Kinesiotherapy/ | 103,934 |
| 23 | exp Physiotherapy/ | 117,278 |
| 24 | exp Diet/ | 452,053 |
| 25 | exp Diet Therapy/ | 434,860 |
| 26 | exp Psychotherapy/ | 316,352 |
| 27 | exp Alternative Medicine/ | 76,326 |
| 28 | exp Psychological Adjustment/ | 10,262 |
| 29 | (exercise* or physical activit* or rehabilitation or training or nutrition* or malnutrition or diet* or optimi#ation).tw. | 2,902,456 |
| 30 | ((psych* or emotion*) adj2 (support* or therap* or intervention* or education* or well-being or wellbeing or resilience or adjustment*)).tw. | 144,746 |
| 31 | OR 21-30 | 3,760,622 |
| 32 | 20 AND 31 | 152,770 |
| 33 | ((multimodal or multi-modal or multidimensional or multi-dimensional or multidisciplinary or multi-disciplinary or multicomponent or multi-component) adj (rehab* or support* or therap* or intervention* or manage*)).tw. | 24,994 |
| 34 | 14 OR 32 OR 33 | 177,686 |
| 35 | exp Patient Compliance/ | 187,924 |
| 36 | exp Health Care Access/ | 101,577 |
| 37 | (adherence or engage* or accept* or motivat* or support* or participation* or feasib* or action* or access* or uptake* or approachability or availab* or compliance or barrier* or attendance or retention or completion or attrition or involvement or awareness).tw. | 9,594,075 |
| 38 | (patient* adj3 (preference* or value* or involvement* or perspective* or assessment*)).tw. | 287,454 |
| 39 | OR 35-38 | 9,834,986 |
| 40 | 11 AND 34 AND 39 | 20,177 |
| 41 | limit 40 to yr="2017 -Current" English Language and humans only. | **9,755** |

**Ovid EMCARE 31^st^ May 2023**

| **Search Number** | **Search** | **Results** |
| --- | --- | --- |
| 1 | exp Neoplasm/ | 578,732 |
| 2 | (cancer* or malignan* or carcino* or adenocarcinoma* or neoplas* or tumo?r* or oncolog*).tw. | 854,772 |
| 3 | exp Radiotherapy/ | 88,013 |
| 4 | exp Chemoradiotherapy/ | 7,601 |
| 5 | exp Antineoplastic Agents/ | 250,089 |
| 6 | exp Chemotherapy/ | 96,371 |
| 7 | exp Adoptive Immunotherapy/ | 2,521 |
| 8 | (chemotherap* or chemorad* or chemo-rad*).tw. | 115,551 |
| 9 | ((radiation or radio* or irradiat*) adj2 (therap* or treatment*)).tw. | 45,074 |
| 10 | (check point inhibitor* or checkpoint inhibitor* or check point blockade or checkpoint blockade).tw. | 7,638 |
| 11 | OR 1-10 | 1,136,110 |
| 12 | exp Preoperative Exercise/ | 121 |
| 13 | prehab*.tw. | 669 |
| 14 | 12 OR 13 | 710 |
| 15 | (pre-operative or preoperative or peri-operative or perioperative or pre-surg* or presurg* or pre-treatment or pretreatment).tw. | 185,140 |
| 16 | exp Preoperative Care/ | 6,889 |
| 17 | exp Perioperative Care/ | 27,195 |
| 18 | exp Preoperative Period/ | 79,578 |
| 19 | exp Perioperative Period/ | 209,771 |
| 20 | OR 15-19 | 339,865 |
| 21 | exp Exercise/ | 138,534 |
| 22 | exp Kinesiotherapy/ | 31,204 |
| 23 | exp Physiotherapy/ | 35,769 |
| 24 | exp Diet/ | 75,444 |
| 25 | exp Diet Therapy/ | 82,735 |
| 26 | exp Psychotherapy/ | 90,842 |
| 27 | exp Relaxation Training/ | 3,930 |
| 28 | exp Coping Behavior/ | 41,699 |
| 29 | exp Psychological Adjustment | 2,752 |
| 30 | (exercise* or physical activit* or rehabilitation or training or nutrition* or malnutrition or diet* or optimi#ation).tw. | 864,693 |
| 31 | ((psych* or emotion*) adj2 (support* or therap* or intervention* or education* or well-being or wellbeing or resilience or adjustment*)).tw. | 66,053 |
| 32 | OR 21-31 | 1,099,174 |
| 33 | 20 AND 32 | 37,122 |
| 34 | ((multimodal or multi-modal or multidimensional or multi-dimensional or multidisciplinary or multi-disciplinary or multicomponent or multi-component) adj (rehab* or support* or therap* or intervention* or manage*)).tw. | 7,647 |
| 35 | 14 OR 33 OR 34 | 44,865 |
| 36 | exp Patient Compliance/ | 42,059 |
| 37 | exp Health Care Access/ | 31,469 |
| 38 | (adherence or engage* or accept* or motivat* or support* or participation* or feasib* or action* or access* or uptake* or approachability or availab* or compliance or barrier* or attendance or retention or completion or attrition or involvement or awareness).tw. | 2,217,044 |
| 39 | (patient* adj3 (preference* or value* or involvement* or perspective* or assessment*)).tw. | 74,322 |
| 40 | OR 36-39 | 2,277,709 |
| 41 | 11 AND 35 AND 40 | 3,706 |
| 42 | limit 41 to yr="2017 -Current" English Language | **1,827** |

**APA PsycINFO (Ovid) 31^st^ May 2023**

| **Search Number** | **Search** | **Results** |
| --- | --- | --- |
| 1 | exp Neoplasms/ | 60,668 |
| 2 | (cancer* or malignan* or carcino* or adenocarcinoma* or neoplas* or tumo?r* or oncolog*).tw. | 96,196 |
| 3 | exp Radiation Therapy/ | 1,450 |
| 4 | exp Chemotherapy/ | 3,581 |
| 5 | exp Antineoplastic Drugs/ | 320 |
| 6 | exp Immunotherapy/ | 8,016 |
| 7 | (chemotherap* or chemorad* or chemo-rad*).tw. | 7,345 |
| 8 | ((radiation or radio* or irradiat*) adj2 (therap* or treatment*)).tw. | 1,941 |
| 9 | (check point inhibitor* or checkpoint inhibitor* or check point blockade or checkpoint blockade).tw. | 71 |
| 10 | OR 1-9 | 107,610 |
| 11 | prehab*.tw | 70 |
| 12 | (pre-operative or preoperative or peri-operative or perioperative or pre-surg* or presurg* or pre-treatment or pretreatment).tw. | 28,940 |
| 13 | exp Exercise/ | 31,884 |
| 14 | exp Physical Therapy/ | 3,555 |
| 15 | exp Diets/ | 20,110 |
| 16 | exp Nutrition/ | 73,649 |
| 17 | exp Psychotherapy/ | 221,186 |
| 18 | exp Cognitive Behavior Therapy/ | 26,714 |
| 19 | exp Mind Body Therapy/ | 374 |
| 20 | exp Relaxation Therapy/ | 3,695 |
| 21 | exp Coping Behavior/ | 54,125 |
| 22 | exp Emotional Adjustment/ | 22,974 |
| 23 | (exercise* or physical activit* or rehabilitation or training or nutrition* or malnutrition or diet* or optimi#ation).tw. | 524,765 |
| 24 | ((psych* or emotion*) adj2 (support* or therap* or intervention* or education* or well-being or wellbeing or resilience or adjustment*)).tw. | 159,260 |
| 25 | OR 13-24 | 159,260 |
| 26 | 12 AND 25 | 945,160 |
| 27 | ((multimodal or multi-modal or multidimensional or multi-dimensional or multidisciplinary or multi-disciplinary or multicomponent or multi-component) adj (rehab* or support* or therap* or intervention* or manage*)).tw. | 3,261 |
| 28 | 11 OR 26 OR 27 | 10,204 |
| 29 | exp Treatment Compliance/ | 17,538 |
| 30 | exp Health Care Access/ | 9,138 |
| 31 | exp Health Care Utilization/ | 17,902 |
| 32 | exp Treatment Barriers/ | 7,083 |
| 33 | (adherence or engage* or accept* or motivat* or support* or participation* or feasib* or action* or access* or uptake* or approachability or availab* or compliance or barrier* or attendance or retention or completion or attrition or involvement or awareness).tw. | 1,957,822 |
| 34 | (patient* adj3 (preference* or value* or involvement* or perspective* or assessment*)).tw. | 26,221 |
| 35 | OR 29-34 | 1,979,082 |
| 36 | 10 and 28 and 35 | 352 |
| 37 | limit 36 to yr="2017 -Current" English Language | **145** |

**AMED (Ovid) 31^st^ May 2023**

| **Search Number** | **Search** | **Results** |
| --- | --- | --- |
| 1 | exp Neoplasms/ | 17,878 |
| 2 | (cancer* or malignan* or carcino* or adenocarcinoma* or neoplas* or tumo?r* or oncolog*).tw. | 24,654 |
| 3 | exp Radiotherapy/ | 747 |
| 4 | exp Antineoplastic agents/ | 5.090 |
| 5 | exp Immunotherapy/ | 120 |
| 6 | (chemotherap* or chemorad* or chemo-rad*).tw. | 2,094 |
| 7 | ((radiation or radio* or irradiat*) adj2 (therap* or treatment*)).tw. | 782 |
| 8 | (check point inhibitor* or checkpoint inhibitor* or check point blockade or checkpoint blockade).tw. | 10 |
| 9 | OR 1-8 | 26,671 |
| 10 | prehab*.tw. | 47 |
| 11 | (pre-operative or preoperative or peri-operative or perioperative or pre-surg* or presurg* or pre-treatment or pretreatment).tw. | 4,439 |
| 12 | exp Preoperative care/ | 400 |
| 13 | 11 OR 12 | 4,439 |
| 14 | exp Exercise/ | 10,669 |
| 15 | exp Exercise therapy/ | 9,600 |
| 16 | exp physical therapy modalities/ | 32,200 |
| 17 | exp Diet/ | 2,108 |
| 18 | exp Nutrition therapy/ | 2,261 |
| 19 | exp Psychotherapy/ | 10,540 |
| 20 | exp Mind body medicine/ | 239 |
| 21 | exp Adaptation psychological/ | 4,404 |
| 22 | (exercise* or physical activit* or rehabilitation or training or nutrition* or malnutrition or diet* or optimi#ation).tw. | 113,768 |
| 23 | ((psych* or emotion*) adj2 (support* or therap* or intervention* or education* or well-being or wellbeing or resilience or adjustment*)).tw. | 6,093 |
| 24 | OR 14-23 | 139,131 |
| 25 | 13 AND 24 | 1,429 |
| 26 | ((multimodal or multi-modal or multidimensional or multi-dimensional or multidisciplinary or multi-disciplinary or multicomponent or multi-component) adj (rehab* or support* or therap* or intervention* or manage*)).tw. | 491 |
| 27 | 10 AND 25 AND 26 | 1,938 |
| 28 | exp Patient compliance/ | 1,135 |
| 29 | exp Health services accessibility/ | 1,297 |
| 30 | (adherence or engage* or accept* or motivat* or support* or participation* or feasib* or action* or access* or uptake* or approachability or availab* or compliance or barrier* or attendance or retention or completion or attrition or involvement or awareness).tw. | 77,059 |
| 31 | (patient* adj3 (preference* or value* or involvement* or perspective* or assessment*)).tw. | 9,816 |
| 32 | OR 28-31 | 83,830 |
| 33 | 9 and 27 and 32 limited to yr="2017 -Current" English Language | **26** |

**CINAHL (EBSCO) 31^st^ May 2023**

| **Search Number** | **Search** | **Results** |
| --- | --- | --- |
| 1 | (MH “Neoplasms+”) | 652,827 |
| 2 | TI (cancer* or malignan* or carcino* or adenocarcinoma* or neoplas* or tumo?r* or oncolog*) OR AB (cancer* or malignan* or carcino* or adenocarcinoma* or neoplas* or tumo?r* or oncolog*) | 663,199 |
| 3 | (MH “Radiotherapy+”) | 41,468 |
| 4 | (MH “Chemoradiotherapy”) | 1,423 |
| 5 | (MH “Antineoplastic Agents+”) | 137,890 |
| 6 | (MH “Chemotherapy Adjuvant+”) | 13,389 |
| 7 | (MH “Immunotherapy+”) | 55,407 |
| 8 | TI (chemotherap* or chemorad* or chemo-rad*) OR AB (chemotherap* or chemorad* or chemo-rad*) | 95,491 |
| 9 | TI (radiation or radio* or irradiat*) adj2 (therap* or treatment*) OR AB ((radiation or radio* or irradiat*) adj2 (therap* or treatment*) | 39,441 |
| 10 | TI (check point inhibitor* or checkpoint inhibitor* or check point blockade or checkpoint blockade) OR AB (check point inhibitor* or checkpoint inhibitor* or check point blockade or checkpoint blockade) | 7,368 |
| 11 | OR 1-10 | 965,003 |
| 12 | (MH “Prehabilitation”) | 297 |
| 13 | TI Prehab* OR AB Prehab* | 643 |
| 14 | 12 OR 13 | 761 |
| 15 | TI (pre-operative or preoperative or peri-operative or perioperative or pre-surg* or presurg* or pre-treatment or pretreatment) OR AB (pre-operative or preoperative or peri-operative or perioperative or pre-surg* or presurg* or pre-treatment or pretreatment) | 125,400 |
| 16 | MH (“Preoperative Care+”) | 24,787 |
| 17 | (MH “Perioperative Care+”) | 62,096 |
| 18 | (MH “Preoperative Period+”) | 7,561 |
| 19 | OR 15-18 | 169,207 |
| 20 | (MH “Exercise+”) | 127,959 |
| 21 | (MH “Therapeutic Exercise+”) | 61,988 |
| 22 | (MH “Physical Therapy+”) | 159,216 |
| 23 | (MH “Diet+”) | 138,414 |
| 24 | (MH “Diet Therapy+”) | 36,255 |
| 25 | (MH “Psychotherapy+”) | 226,294 |
| 26 | (MH “Mind Body Techniques+”) | 46,735 |
| 27 | (MH “Adaptation, Psychological+”) | 40,614 |
| 28 | (MH “Coping+”) | 42,596 |
| 29 | TI (exercise* or physical activit* or rehabilitation or training or nutrition* or malnutrition or diet* or optimi?ation) OR AB (exercise* or physical activit* or rehabilitation or training or nutrition* or malnutrition or diet* or optimi?ation) | 719,892 |
| 30 | TI (psych* or emotion*) N2 (support* or therap* or intervention* or education* or well-being or wellbeing or resilience or adjustment*) OR AB ((psych* or emotion*) N2 (support* or therap* or intervention* or education* or well-being or wellbeing or resilience or adjustment*) | 67,258 |
| 31 | OR 20-30 | 1,191,639 |
| 32 | 19 AND 31 | 20,414 |
| 33 | TI (multimodal or multi-modal or multidimensional or multi-dimensional or multidisciplinary or multi-disciplinary or multicomponent or multi-component) W1 (rehab* or support* or therap* or intervention* or manage*) AND AB (multimodal or multi-modal or multidimensional or multi-dimensional or multidisciplinary or multi-disciplinary or multicomponent or multi-component) W1 (rehab* or support* or therap* or intervention* or manage*) | 8,480 |
| 34 | 14 OR 32 OR 33 | 29,061 |
| 35 | (MH “Attitude to Medical Treatment”) | 1,950 |
| 36 | (MH “Treatment Refusal+”) | 6288 |
| 37 | (MM “Medication Compliance”) | 12,756 |
| 38 | (MH “Health Services Accessibility+”) | 104,578 |
| 39 | TI (adherence or engage* or accept* or motivat* or support* or participation* or feasib* or action* or access* or uptake* or approachability or availab* or compliance or barrier* or attendance or retention or completion or attrition or involvement or awareness) OR AB (adherence or engage* or accept* or motivat* or support* or participation* or feasib* or action* or access* or uptake* or approachability or availab* or compliance or barrier* or attendance or retention or completion or attrition or involvement or awareness) | 8,449 |
| 40 | TI (patient* N3 (preference* or value* or involvement* or perspective* or assessment*) OR AB (patient* N3 (preference* or value* or involvement* or perspective* or assessment*) | 79,804 |
| 41 | OR 35-40 | 1,736,179 |
| 42 | 11 AND 34 AND 41 | **1,500** |

**Cochrane (CENTRAL) 31^st^ May 2023**

| **Search Number** | **Search** | **Results** |
| --- | --- | --- |
| 1 | MeSH descriptor: [Neoplasms] explode all trees | 111,266 |
| 2 | MeSH descriptor: [Radiotherapy] explode all trees | 10,315 |
| 3 | MeSH descriptor: [Antineoplastic Agents] explode all trees | 16,553 |
| 4 | MeSH descriptor: [Chemoradiotherapy] explode all trees | 2216 |
| 5 | MeSH descriptor: [Immunotherapy, Adoptive] explode all trees | 153 |
| 6 | (cancer OR malignan* OR carcino* OR adenocarcinoma* OR neoplas* OR tum*r OR oncolog*):ti,ab,kw | 263,590 |
| 7 | (chemotherap* OR chemorad* OR chemo-rad*):ti,ab,kw | 94,765 |
| 8 | (radiation OR radio* OR irradiat*) NEAR/2 (therap* OR treatment*) | 27,119 |
| 9 | "check point inhibitor*" OR "checkpoint inhibitor*" OR "check point blockade" OR "checkpoint blockade" | 1137 |
| 10 | OR 1-9 | 291,454 |
| 11 | MeSH descriptor: [Preoperative Exercise] explode all trees | 75 |
| 12 | (prehab*):ti,ab,kw | 632 |
| 13 | 11 OR 12 | 645 |
| 14 | MeSH descriptor: [Preoperative Care] explode all trees | 6751 |
| 15 | MeSH descriptor: [Preoperative Period] explode all trees | 763 |
| 16 | MeSH descriptor: [Perioperative Care] explode all trees | 14,480 |
| 17 | MeSH descriptor: [Perioperative Period] explode all trees | 12,241 |
| 18 | (pre-operative OR preoperative OR peri-operative OR perioperative OR pre-surg* OR presurg* OR pre-treatment OR pretreatment):ti,ab,kw | 87,714 |
| 19 | OR 14-18 | 101,847 |
| 20 | MeSH descriptor: [Exercise] explode all trees | 38,145 |
| 21 | MeSH descriptor: [Exercise Therapy] explode all trees | 19,513 |
| 22 | MeSH descriptor: [Physical Therapy Modalities] explode all trees | 35,620 |
| 23 | MeSH descriptor: [Diet] explode all trees | 25,632 |
| 24 | MeSH descriptor: [Diet Therapy] explode all trees | 7,719 |
| 25 | MeSH descriptor: [Psychotherapy] explode all trees | 33,286 |
| 26 | MeSH descriptor: [Mind-Body Therapies] explode all trees | 8723 |
| 27 | MeSH descriptor: [Relaxation Therapy] explode all trees | 2450 |
| 28 | MeSH descriptor: [Adaptation, Psychological] explode all trees | 6340 |
| 29 | (exercise OR "physical activit*" OR rehabilitation OR training OR nutrition* OR malnutrition* OR diet* OR optimi*ation):ti,ab,kw | 349,949 |
| 30 | ((psych* OR emotion*) NEAR/2 (support* OR therap* OR intervention* OR education* OR well-being OR wellbeing OR resilience OR adjustment*)):ti,ab,kw | 56,653 |
| 31 | OR 20-30 | 418,016 |
| 32 | 19 AND 31 | 15,756 |
| 33 | ((multimodal OR multi-modal OR multidimensional OR multi-dimensional OR multidisciplinary OR multi-disciplinary OR multicomponent OR multi-component) NEAR/2 (rehab* OR support* OR therap* OR intervention* OR manage*)):ti,ab,kw | 5670 |
| 34 | 13 OR 32 OR 33 | 21,472 |
| 35 | MeSH descriptor: [Patient Compliance] explode all trees | 15,185 |
| 36 | MeSH descriptor: [Health Services Accessibility] explode all trees | 1495 |
| 37 | (adherence OR engage* OR accept* OR motivat* OR support* OR participation* OR feasibil* OR action* OR access* OR uptake* OR approachability OR availab* OR compliance OR barrier* OR attendance OR retention OR completion OR attrition OR involvement OR awareness):ti,ab,kw | 557,284 |
| 38 | (patient NEAR/3 (preference* OR value* OR involvement* OR perspective* OR assessment*)):ti,ab,kw | 32,210 |
| 39 | OR 35-38 | 573,269 |
| 40 | 10 AND 34 AND 39 with publication year from 2017 to 2023, In Trials | **1239** |

**Pedro**

| Prehab* AND cancer* | 64 |
| --- | --- |
| “preoperative exercise” AND cancer* | 20 |
| “pre-operative exercise” AND cancer* | 7 |
| Imported into Endnote | **91** |

| **Database** | **Number of References** |
| --- | --- |
| Medline (Ovid) | 4248 |
| EMBASE (Ovid) | 9755 |
| Ovid Emcare | 1827 |
| APA PsycINFO (Ovid) | 145 |
| AMED (Ovid) | 26 |
| CINAHL (EBSCO) | 1500 |
| Cochrane CENTRAL | 1239 |
| Pedro | 91 |
| TOTAL | **18,831** |
| Duplicates identified | 7116 |
| New Total | **11,715** |
