# Supplementary material for: Access, acceptance and adherence to cancer prehabilitation: a mixed-methods systematic review
Source: J Cancer Surviv. 2024 May 6;19(6):1895–923. doi: 10.1007/s11764-024-01605-3 (PMC12546383; doi:10.1007/s11764-024-01605-3)
Supplement: Supplementary file 2 — Supplementary file2 (DOCX 99.3 KB) [file 11764_2024_1605_MOESM2_ESM.docx]

| **Study author**  **Year** | **Aim(s)** | **Summary of key findings main findings** |
| --- | --- | --- |
| Prepare ABC Trial Collaborative [28]  2021 | To confirm feasibility of site set-up and patient recruitment, acceptability of the interventions and patient adherence to hospital-supervised and home-supported exercise. | **Adherence**  Adherence in hospital supervised groups was measured by: preop: exercise sessions attended and post op supervised booster sessions. Preoperatively at least 57% attended six session; at least 73% attended four sessions attended and at least 95% attended one or more sessions. Adherence in home supported exercise programme measured by preop: patient engagement with weekly telephone support Preoperatively at least 70% attended two or more telephone support sessions and 85% at least one telephone support session.  *Barriers to adherence*  Having to travel to hospital for supervised exercise preoperatively.  **Acceptance**  Health professionals experienced logistical difficulties in the shape of booking rooms for exercise, telephone support and securing use of exercise equipment. |
| Agasi-Idenburg 2020 [32] | To investigate the barriers, facilitators, and preferences for preoperative exercise programs in older patients scheduled for CRC surgery. | **Access**  *Facilitators of access*  Prehabilitation healthcare professionals (physiotherapists) emphasised the importance of physician recommendations.  *Barriers to access*  Prehabilitation healthcare professionals (physiotherapists) reported that barriers to prehabilitation access included patients’ physical limitations due to co-morbid conditions and pain; worry and anxiety; competing priorities prior to treatment; volume of hospital appointments and the limited time between completion of neoadjuvant treatment and surgery.  **Adherence**  *Facilitators of adherence*  Prehabilitation healthcare professionals (physiotherapists) emphasised the importance of social and peer support and prehabilitation interventions which are personalised to address individual need and delivered in close proximity to individuals’ homes. |
| Argudo [29]  2021 | To investigate the feasibility and tolerability of a 5-week preoperative high-intensity interval training program after NAT, and to assess the potential effects of the training protocol on exercise capacity, muscle function, and health-related quality of life (HRQL) | **Adherence**  Patients attended a mean of 19.4 (standard deviation 6.4) of the 25 scheduled supervised exercise sessions, and 27 out of 33 (81.8%) patients achieved the predefined level of attendance (a minimum of 15 of the 25 scheduled sessions).  *Barriers to adherence*  Transport problems and outpatient appointments. |
| Banerjee  2019 [33] | To investigate the perspectives and experiences of bladder cancer patients who participated in a programme of vigorous intensity aerobic interval exercise prior to radical cystectomy. | **Access**  *Facilitators of access*  Facilitators of access included motivation, previous engagement in exercise, the opportunity to improve post-operative outcomes and family members’ encouragement to participate in the preoperative exercise program.  *Barriers to access*  Travel and parking.  **Adherence**  *Facilitators of adherence*  Receiving appropriate supervision, objective measures of fitness improvements and support from healthcare professionals and family members were strong motivators for maintaining participation in prehabilitation. Prior exercise experience gave some participants the confidence to push themselves during the exercise sessions. Anticipated and/or objective improvements in cardiopulmonary fitness during the exercise program motivated individuals to continue attending. Some spouses or a family member accompanied participants to the exercise training facility, and even took part, while others encouraged participants to undertake exercise at home. Supervised exercise provision before surgery was a key aspect of participants maintaining attendance at the exercise program.  *Barriers to adherence*  The physical discomfort of bike seat.  **Acceptance**  Participants were positive about the exercise program structure and design and their relationship with, and support received from the exercise instructor. They found sessions enjoyable, helpful and interesting. |
| Beck  2021 [45] | To understand perspectives on and acceptability of prehabilitation among patients undergoing complex abdominal cancer surgery | **Adherence**  Participants showed adherence to the recommendations listed in the leaflet as more than 50% of the participants adhered to over 75% of the individual recommendations.  *Facilitators of adherence*  Facilitators of adherence to prehabilitation included harbouring a positive attitude toward prehabilitation and recognising it could be beneficial. The chance to personalise prehabilitation by tailoring the recommendations according to their needs, resources, everyday lives and preferences -meant prehabilitation was realistic, manageable and amenable to change, especially when individuals were challenged by their physical and /or psychological condition. The opportunity to influence their recovery by strengthening their bodies was perceived as a motivating and distracting force. Recording actions in the leaflet, knowing that a health professional would go through their notes, and for some patients, use it in the interviews, motivated them to perform prehabilitation-related actions. While freedom and flexibility to engage in prehabilitation was valued, professional support and collaboration was still needed. Home-based prehabilitation felt safer and was easier to fit into individuals’ everyday lives.  *Barriers to adherence*  Limited time pre operatively meant that prehabilitation had a lower priority for participants. Their focus was on having their surgery completed as soon as possible and engaging in meaningful everyday activities and practicalities including those which would prepare them for their recovery period. Putting their life in order was a priority prior to surgery. Most patients had symptoms preoperatively which limited their ability to engage in prehabilitation activities and general recommendations in a leaflet could be irrelevant on an individual level.  **Acceptance**  Patients appreciated the freedom to tailor their prehabilitation to meet their needs and to engage with prehabilitation at home as they could fit in the prehabilitation according to their resources, everyday lives and preferences and live their lives as normally as possible prior to surgery. |
| Beck  2022 [34] | To investigate the experiences,  thoughts, and feelings that underlie and influence actions or the lack of actions in relation to prehabilitation among cancer patients due to undergo major abdominal surgery | **Adherence**  *Facilitators of adherence*  Earlier access to prehabilitation when the preoperative period was short. Home based prehabilitation offered greater flexibility to accommodate prehabilitation into everyday lives and engendered a sense of control while facility based prehabilitation offered healthcare professional support and supervision and access to peer support which was motivating. Family members provided motivational support. Daily documentation of prehabilitation actions completed was motivating, enabled participants to set personal goals and generated a sense of calm and success.  *Barriers to adherence*  Personal priorities prior to surgery including spending time with families, living their lives, putting affairs in order and preparing for surgery and recovery meant time for prehabilitation was hard to find. Facility based prehabilitation was perceived as disruptive to participants’ everyday lives in advance of surgery as it was time consuming and set limits on time spent with families. Physical symptoms and psychological status also set limits on the ability to engage in prehabilitation, particularly in the absence of professional support and some felt that prehabilitation was irrelevant, either because they thought they were too fit or did not feel ill. Concern was expressed about being a burden to other family members while pressure from health professionals could result in resistance.  **Acceptance**  Participants appreciated the home-based recommendations in the leaflet. Home was perceived as being a safe space when experiencing physical symptoms / anxiety. On a practical level home based prehabilitation enabled participants to fit prehabilitation into their everyday lives, work and families and created opportunities for family participation in physical activities. |
| Beck  2021 [46] | To investigate what patients with cancer who were due to undergo major abdominal surgery (CRS HIPEC) actually were able to do when provided with preoperative, home-based, multimodal recommendations presented in a leaflet, instead of taking part in a standardised programme. | **Adherence**  53 (73%) of the 73 participants who received the leaflet returned a completed leaflet, representing their prehabilitation activities. Over half adhered to more than 75% to the individual recommendations about exercise, nutrition and relaxation.  *Facilitators of adherence*  Making notes and ticking boxes helped participants to systematise their preparation and motivated them to complete activities. General recommendations provided participants with the freedom to choose between different small, realistic and manageable activities, allowing them to tailor their preparation according to their activity level, everyday lives, preferences and individual needs. If directly involved in prehabilitation family members could facilitate adherence.  *Barriers to adherence*  Physical symptoms, family gatherings, work, anxiety, practical activities, the weather and family illness were perceived as impediments to engaging in prehabilitation. General recommendations that could be perceived as irrelevant, the absence of health professional and social support and family conflict were also barriers to adherence.  **Acceptance**  The leaflet was a motivator for action as the recommendations were perceived as realistic and manageable. |
| Bingham  2023 [12] | To explore mechanisms promoting feasibility and acceptability of a MCPP  from patients and professionals perspectives exploring planning, development and implementation. | **Access**  *Facilitators of access*  Endorsement from healthcare professionals, understanding the purpose of prehabilitation and believing engagement in the prehabilitation can improve patient outcomes.  *Barriers to access*  Some healthcare professionals perceived that some forms of exercise prehabilitation, for example HIIT, is unrealistic for some patients, particularly those with co-morbidities and who are older. They also noted that introducing patients to prehabilitation when receiving their cancer diagnosis could function as a barrier as patients could be emotionally overwhelmed with information. Insufficient staff capacity to deliver equitable services.  **Adherence**  *Facilitators of adherence*  From the perspective of healthcare professionals, timing of prehabilitation, endorsement of exercise from the clinical team, appropriately prepared prehabilitation healthcare professionals, suitably resourced prehabilitation teams and sustained funding were important. For patients, the motivational and support and encouragement of prehabilitation healthcare professionals who were available and committed was valued, as were family and peer support.  *Barriers to adherence*  Short timeframe between diagnosis and treatment when patients also had to attend to personal affairs and attend other clinics.  **Acceptance**  Patients viewed the tailored and personalised exercise programme as being acceptable as it was patient-centred, varied and comprised of achievable attributes, including indoor and outdoor options. |
| Bradley  2023 [13] | Examine the feasibility, uptake, participation, and clinical  outcomes from the Greater Manchester Prehab4Cancer (P4C) programme for lung cancer patients with planned surgical resection. | **Access**  *Facilitators of access*  Perceived benefits of prehabilitation; influence of clinical team. Collaboration with an existing community leisure service infrastructure and smooth referral pathways from NHS care to community leisure teams.  *Barriers to access*  Imminent surgery and being unwell.  **Adherence**  Of patients who attended a first assessment, 64.3% (n =180/280) completed the prehabilitation phase with a median number of sessions completed of 6 (IQR: 4-9). |
| Brady  2020 [35] | The aim of the project was to improve service provision of pre-treatment SLT assessment and information counselling for patients undergoing radiation treatment for HNC. | **Access**  *Facilitators of access*  Awareness and understanding of the role and focus of speech and language therapists (SLT) prior to attending the SLT clinic and the rationale for the appointment. Flexible appointment system and joint appointments.  *Barriers to access*  No awareness and understanding of the role and focus of speech and language therapists (SLT) and the rationale for the appointment. Previous experiences of SLT.  **Adherence**  *Facilitators of adherence*  Confidence in the professional team, consistency in messaging from the team and information provided in bite size chunks which met individual needs and did not overload them. Enjoyed routine of manageable exercise and understood benefits of completing exercises.  *Barriers to adherence*  Information overload can be overwhelming and dislike of exercise. |
| Brahmbhatt  2020 [58] | To assess the feasibility and acceptability of an individualized, home-based prehabilitation intervention prior to breast cancer surgery. | **Adherence**  Average adherence to the minimum range of the aerobic exercise prescription was 142.22 ± 82.66% and adherence to the resistance training prescription was 114.44 ± 38.26%. Most participants exceeded their prescribed level. This was because most participants were exercising beyond the lower end of their exercise prescription range. Of the 17 participants that provided adherence data, 13 (76%) were considered adherent to their prescription (i.e., completed >70% of exercise volume prescribed for each session). Two participants partially adhered to their prescription (i.e., completed <70% of their prescribed exercise volume in some sessions) and two participants were non-adherent i.e., completed <70% of their prescribed exercise volume in all sessions).  *Facilitators of adherence*  Portability of the resistance bands enabled some individuals to continue exercise routine while travelling. The individualised intervention was perceived as convenient and regardless of previous physical activity experience was described as being easy to follow. In-person exercise instruction with an oncology trained healthcare professional and feedback was helpful and increased how comfortable participants felt with being able to exercise on their own at home. Weekly phone conversations. Exercise logs enabled participants to track their adherence and created a sense of accountability and control, which was augmented by the structure of the exercise prescription.  *Barriers to adherence*  Motivation, the weather, insufficient time in the preoperative period.  **Acceptance**  Eleven participants completed the participant satisfaction survey at the final study assessment. Ten (90.9%) found the exercise manual helpful, and eight (72.7%) said they were able to complete all the exercises prescribed to them. On average, participants rated the program 8.6 out of a score of 10, with 0 being the lowest and 10 being the highest score possible. All respondents indicated that they planned to continue regular exercise, would recommend the program and believed that prehabilitation helped them recover after surgery and felt better during subsequent treatment(s) because of it. |
| Burden  2017 [30] | To determine if pre-operative ONS with dietary advice, compared with dietary advice only, can reduce post-operative infections in people prior to surgical re-section for colorectal cancer who have previously lost weight | **Adherence**  Two-thirds of the participants managed more than 75% of the recommended dose. Most participants who were randomized to the ONS were able to consume some of the drink, and a 71% adherence rate was recorded. Of the 53 participants in the intervention arm 39 (74%) returned their self-reported supplement diaries. Twenty nine (74%) self-reported taking all the supplements daily (two cartons). Two (5%) participants reported that they managed one and a half cartons, 3 (8%) participants reported that they managed one carton, 3 (8%) managed half a carton, and 2 (5%) participants reported that they did not consume any of the ONS.  *Barriers to adherence*  Unpalatability and intolerance of ONS, nausea, abdominal discomfort and diarrhoea. |
| Catho  2021 [31] | To identify determinants of non-completion of an H-RP and the factors associated with medical events occurring 30 days after hospital discharge. | **Adherence**  Twenty participants (48%) completed the whole programme.  *Barriers to adherence*  Living alone, polypharmacy and a long delay before starting the programme. |
| Collaco  2021 [36] | To explore patients and healthcare professionals views and experiences of a pre- and post-operative rehabilitation intervention (SOLACE), for patients undergoing surgery for early-stage lung cancer. | **Access**  *Facilitators of access*  Healthcare professionals perceived that clinicians’ endorsement, information leaflets and referrals from the wider healthcare team at the right time were pivotal in supporting access.  *Barriers to access*  Healthcare professionals’ busy workloads. Insufficient awareness of the service among patients. Patients’ perceptions of being fit enough, living too far away to attend, having mobility issues, imminent surgery and making lifestyle changes which may incur financial expenditure all acted as barriers to uptake of prehabilitation.  **Adherence**  *Facilitators of adherence*  Prehabilitation professionals’ support and encouragement to engage with the programme. Personalised approach and a supportive peer group environment which provided emotional support and a space to share treatment experiences. Desire to improve pre-treatment fitness and optimise recovery.  *Barriers to adherence*  Absence of healthcare professional facilitation and supervision meant sustaining motivation to continue was difficult.  **Acceptance**  All patient participants reported positive experiences of the SOLACE service. The types of support they valued included receiving responses to queries and concerns, the provision of tailored support and advice, and being signposted to other support services, as necessary. Patients recognised the importance of personalised care and the SOLACE team creating a safe and comfortable space to discuss concerns. Patient participants who had attended rehabilitation classes reported benefits of a supportive peer group environment and knowing they were all “in it together”. The classes provided emotional support and opportunities to talk through experiences of surgery and related side effects. Patient participants reported marked improvements in their recovery from attending rehabilitation classes, including increased exercise confidence and improvements in mobility and breathing, increasing their desire to be more active. Some participants felt that the programme had helped them to achieve their fitness goals. This, in turn, led to improvements in self-esteem, motivation, well-being and exercise maintenance. |
| Cooper  2022 [37] | To identify factors influencing uptake, engagement and adherence to the ChemoFit intervention and to establish whether it was acceptable and feasible to use. | **Access**  *Facilitators of access*  Motivated by increased chance of good recovery, shorter hospital stay and survival if fitness was improved. Motivational support of family and friends and a desire to play part in recovery and reduce the burden on NHS.  *Barriers to access*  COVID-19 related lockdown measures created barriers to access. Inability to retain information at initial consultation due to feeling overwhelmed as also given information about diagnosis and treatment.  **Adherence**  Adherence to the intervention was good. exercises were completed ~70% of the time, there was 99% compliance with recording physical activity and exercise, and 100% compliance with a weekly telephone consultation.  *Facilitators of adherence*  Tracking activity and exercise levels in a diary and the identification of realistic strategies to keep going in the face of adversity was a motivating force and provided an incentive to keep going with the programme. The ability to fit physical activity and exercise into participants’ routines at home, engage family members living in the home, tailor the intervention to their individual needs and preferences, while achieving goals was a distraction, helped improve fitness and avoid time and expense associated with travelling. Completing the intervention at home enabled participants to feel more comfortable to complete the exercises in their own environment, or an environment of their choice, on their terms and eliminated competitiveness and self-consciousness. Ongoing support from clinical team.  *Barriers to adherence*  Participants reported difficulty continuing with activity and exercise when unwell or undergoing treatment. Unattainable goals meant some gave up. Some exercises and equipment was challenging to master and maintain, e.g., static bike, strength exercises and left participants feeling unwell. Comorbidities such as arthritis impacted on performance. Barriers to walking made achieving steps targets difficult, for example poor weather, boredom with same circuits / routes. Insufficient peer support.  **Acceptance**  The intervention was acceptable to participants. Many of the participants consulted attributed their recovery and survival from cancer directly to the intervention. A home-based programme facilitated integration of physical activity and exercise into participants’ daily routine, they could involve partners and friends, and they could engage at a time most convenient to them. The flexibility of the intervention meant participants could adapt it to suit their own capabilities and how they were feeling on a given day. This was considered important because participants reported not wanting the additional pressure of having to keep up with others. |
| Crowe  2022 [47] | To evaluate the impact of a new multidisciplinary allied health prehabilitation service in haematologic cancer patients receiving high-dose chemotherapy with autologous stem cell transplant (AuSCT). | **Access**  *Barriers to access*  Distance from home.  **Adherence**  Eighteen percent (n=21) of patients attended supervised group exercise class at the hospital, and on average attended 5.7 group exercise sessions during their prehabilitation period.  *Barriers to adherence*  Change in treatment plan. |
| Daun  2022 [38] | To understand patient and HCP perspectives on the role of multiphasic exercise prehabilitation. | **Access**  *Facilitators of access*  Healthcare professionals perceived that the possibility of prehabilitation may cultivate a sense of hope for the future among patients. Appropriate timing of the prehabilitation offer to promote access. Clinician endorsement and sustained funding.  *Barriers to access*  Patients may be overwhelmed pre surgery. Culture in clinical environments and insufficient funding and administrative and policy level support. Limited education and support for patients.  **Adherence**  *Facilitators of adherence*  Engaging in exercise was perceived as important for recovery, survival and overall health status. Individualised programmes tailored to preferences and accessibility and involving exercise specialists. Education and access to relevant resources.  **Acceptance**  Assessment of patient reported outcomes, physical function, and mobilization were acceptable across the HNC surgical timeline. Activity trackers were acceptable to patients and valued. |
| Deftereos  2022 [59] | To analyse the implementation of a standardised nutrition care pathway for UGI cancer surgery into clinical practice from the perspectives of dietitians, multi-disciplinary team (MDT) members and patients. | **Access**  *Facilitators of access*  Co-location of dieticians in the clinic.  *Barriers to access*  Initial consultations often took place after initial surgical and oncological consultations and a long day in the clinic when patients could be tired and overwhelmed.  **Adherence**  *Facilitators of adherence*  Dietician-patient relationship. Increased dietician involvement pre surgery and flexible approach to intervention delivery and preparedness to individualise care to meet needs and use digital technology when patients unable to attend the clinic. Accessible and relevant information.  *Barriers to adherence*  Insufficient resource and dedicated dietician time to deliver intervention.  **Acceptance**  The nutritional care pathway was perceived as moderately acceptable by dieticians. Results from the patient satisfaction survey indicated that dietetic support was highly rated both overall, and in each section (perceived health benefits, staff presentation and interpersonal skills, expectations and written materials). |
| Deftereos  2021 [48] | To determine the type and frequency of preoperative dietetics intervention and nutrition support received, factors associated with receipt of preoperative dietetics intervention. | **Access**  *Facilitators of access*  Malnutrition; weight loss, neo-adjuvant therapy. Oesophageal and gastric cancer and residing in metropolitan area.  *Barriers to access*  Pancreatic surgery due to short time frame to surgery. |
| Drummond  2022 [49] | To document the implementation of a multi-modal teleprehabilitation for cancer patients undergoing elective thoracic and abdominal cancer resection surgery and patients' experience of the program | **Access**  *Facilitators of access*  Virtual option of the intervention and loan of equipment when individuals did not have personal devices: training watches and tablets.  **Adherence**  *Facilitators of adherence*  Support via digital platform, healthcare professionals and family members who exercised with them.  *Barriers to adherence:*  Insufficient digital literacy and technological difficulties. Disease progression. Commencement of adjuvant therapy. Disinterest.  **Acceptance**  The intervention was well received by patients. It was perceived as useful and beneficial on physical fitness, mental health, symptoms, social health and diet. It allowed for greater flexibility in clinical scheduling and exercise interventions. |
| Ferreira  2018 [88] | To better understand patients' perspectives of prehabilitation and to identify factors related to programme adherence | **Access**  *Facilitators of access*  Motivation to be physically prepared for surgery and doctors’ recommendations.  **Adherence**  Adherence rate was 93%.  *Facilitators of adherence*  Patients were in good physical and mental health which enabled participation in physical activity without major limitations or special care. Perception that exercise was important and engagement in physical activity prior to prehabilitation. Supportive team especially weekly follow up telephone calls from the exercise instructor.  *Barriers to adherence*  Transport, parking, cost of parking, making transport arrangements. Time. Programme was perceived to be too challenging.  **Acceptance**  The majority of patients felt supported by the program, the main contributor being the multidisciplinary team. They reported enjoyment with their prehabilitation experience especially the exercise program and supervised training sessions. All patients perceived improvements and most intended to continue exercising. Resistance training was ranked as the most challenging exercise component, followed by aerobic training. Despite this, aerobic training was also ranked as the most enjoyed exercise component. |
| Ferreira  2021 [72] | To assess the feasibility of delivering a novel four-week multimodal prehabilitation intervention combining a mixed-nutrient supplement with structured exercise training and relaxation-strategies for patients with lung cancer awaiting surgical resection | **Adherence**  Adherence to the prescribed intensity of the supervised exercise program was 84.1% (SD 23.1). Self-reported adherence to the home-based exercise program was 88.2% (SD 21) and to the nutritional supplement, 93.2% (SD 14.2).  *Barriers to adherence*  Barriers to the nutritional intervention included digestive issues and forgetting to take the supplement. Mental health problems. Earlier surgery than originally planned. |
| Franssen  2022 [73] | To investigate whether a home-based and tele-monitored prehabilitation program (tele-prehabilitation) is feasible in high-risk patients scheduled for colorectal cancer surgery and to evaluate patient experiences and changes in pre-operative aerobic fitness before and after the tele-prehabilitation program. | **Access**  *Facilitators of access*  Clear understanding of the aim of the intervention and its perceived usefulness.  **Adherence**  All participants completed the tele-prehabilitation program. Adherence with regard to the exercise program’s frequency, intensity, and time was respectively 91%, 84%, and 100%.  *Facilitators of adherence*  Motivation to engage in exercise at home. Perceived exercise as useful, enjoyable and not overly time consuming. Weekly telephone support.  *Barriers to adherence*  Reluctance to exercise prior to surgery.  **Acceptance**  The intervention was perceived as user-friendly and acceptable. |
| Halliday  2021 [74] | To establish whether adherence to a personalised exercise prescription and the amount of physical activity (PA) completed during prehabilitation are related to cardiorespiratory fitness and the incidence of post-operative pneumonia | **Adherence**  *Facilitators of adherence*  Higher baseline fitness.  *Barriers to adherence*  Neoadjuvant chemotherapy prior to surgery, disease progression, deteriorating co-morbidities. |
| Hogan  2019 [39] | To explore enablers and barriers for patients of overall compliance with preoperative  oral nutrition supplements in patients undergoing pelvic exenteration surgery for cancer. | **Adherence**  Twelve of the 20 participants were unable to consume the total amount of the recommended dose. Overall adherence ranged from 1-15 and the median amount of nutrition supplements consumed was 12. In the immunonutrition group, compliance ranged from 2-15 and the median amount of nutrition supplements consumed was 11.5. In the standard polymeric supplement group, compliance ranged from 1-15 and the median amount of nutrition supplements consumed was 12.  *Facilitators of adherence*  Well nourished. Flavour and volume of supplements. Motivation to consume supplements.  *Barriers to adherence*  Disliked flavour of immunonutrition supplements. Volume and texture of supplements made them difficult to consume, particularly if experiencing nausea, anorexia or early satiety. Malnourished at outset. |
| Karlsson  2019 [75] | To evaluate the feasibility of a preoperative, supervised home-based physical exercise program at a high level of estimated exertion, in older people undergoing colorectal cancer surgery in Sweden. | **Access**  *Facilitators of access*  Home-based prehabilitation.  *Barriers to access*  Timing in advance of surgery.  **Adherence**  The median number of supervised sessions attended was 6 (range 4–8) out of a median of 6 planned sessions (range 5–8) over a 17-day exercise period. Compliance with the supervised sessions was 97%. A median of 19 inspiratory muscle training, 6 functional strength, and 8.5 aerobic sessions were reported.  *Facilitators of adherence*  Flexibility of programme. Feeling safe. Home based exercise was motivating and comfortable. Healthcare professional support meant participants felt cared for.  *Barriers to adherence*  Application in practice could be challenging due to time limitations and healthcare professional availability. Rescheduling of surgery. Patients’ other commitments. Some patients find using the inspiratory muscle trainer difficult. Time consuming.  **Acceptance**  Participants generally found the intervention acceptable in terms of information given, the intervention’s content and meaningfulness and the intensity of the supervised exercise. |
| Lawson  2021 [76] | Feasibility and preliminary outcome data on the effects of a 4-week multimodal prehabilitation intervention on muscle characteristics and dietary intake of surgical lung cancer patients. | **Adherence**  Adherence to exercise (86%) and nutritional supplements was high (93%).  *Barriers to adherence*  Mental health problems. Refusal to engage in exercise programme, rescheduling of surgery. Gastro-intestinal problems related to nutritional supplements. |
| Low  2020 [60] | To develop and test a mobile technology-supported intervention to reduce sedentary behaviour before and after cancer surgery, and to evaluate the usability and feasibility of the intervention. | **Adherence**  *Facilitators of adherence*  Ease of digital technology used in the intervention. Smart watch prompted physical activity.  *Barriers to adherence*  Low levels of digital literacy. Other commitments. Connectivity issues. Insufficient personalisation.  **Acceptance**  Moderate acceptability of the intervention was identified. There was some dissatisfaction with the aesthetics of smart watches. |
| Machado  2023 [77] | To determine the feasibility of a homebased exercise program (HBEP) in lung cancer patients undergoing surgical treatment | **Access**  *Facilitators of access*  Home based exercise programme.  **Adherence**  93% completed the home based exercise intervention and post exercise assessment. All participants returned the exercise diaries. A total of 238 exercise sessions were completed out of 224 sessions prescribed, corresponding to a median attendance rate of 100% [IQR 93–107]. Patients completed a median of 9 sessions of aerobic exercise [IQR 8–10] and 7 sessions of resistance exercise [IQR 6–8], corresponding to a median attendance rate of 100% [IQR 89–100] and 100%[IQR 100–117], respectively.  *Facilitators of adherence*  Individualised approach with adjustments to exercise prescription to mitigate the impact of symptoms such as pain. Self-efficacy to engage in prehabilitation exercises.  *Barriers to adherence*  Bad weather, insufficient time, foot pain, comorbidities such as arthritis. Refusal to have surgery.  **Acceptance**  Patients perceived the intervention as highly acceptable, with a median average score of 4.9 [range: 4.4–5], which exceeded the pre-defined study target of an average acceptability score of 4. They perceived the intervention as beneficial in their preparation for surgical treatment (median score of 5 [range: 4–5]). |
| Macleoud  2018 [61] | To assess the practical aspects of delivering and evaluating a lifestyle intervention programme (TreatWELL) for patients with CRC undergoing potentially curative treatment. | **Adherence**  Total median intervention delivery by lifestyle counsellors (LCs) was 5 hours 29 mins. LCs reported that patient engagement was high, with 93%–100% being at least ‘fairly engaged’ at all stages.  *Facilitators of adherence*  Participation was a distraction. Support from the LCs. Pedometers were helpful. Prior enjoyment of walking and previous experience of weight loss programmes. Supportive family members who encouraged adherence to healthy eating and sometimes participated in activity. Cancer diagnosis acted as a motivator to engage with the intervention to be in best position for surgery and facilitate recovery. Progress monitoring was a motivator and was enjoyed.  *Barriers to adherence*  Short time period between diagnosis and surgery when patients had other commitments. Feeling too unwell to engage with physical activity. Comorbidities. Insufficient awareness of the intervention among NHS staff.  **Acceptance**  Consultants’ endorsement was reassuring. The amount of contact, and the balance between visits and telephone calls, appeared acceptable, and the provision of home visits was particularly appreciated. |
| Mawson  2021 [62] | To assess the acceptability of the intervention through qualitative interviews and retention rates during the study. | **Access**  *Barriers to access*  Location and distance of venue for intervention. Travelling. Feeling fatigued when living with myeloma and unable to manage other everyday activities.  **Adherence**  *Facilitators of adherence*  Physiotherapy support and detailed instructions at an appropriate level and individualised.  *Barriers to adherence*  Treatment commenced earlier than originally planned. Timing of classes. Additional visits to the hospital. Challenges getting to the gym.  **Acceptance**  Patients reported improved confidence and overall mental health and wellbeing. |
| McCourt  2023 [40] | To explore the experiences of participants who took part in the PERCEPT myeloma pilot trial in order to aid the design of a fully powered RCT. | **Adherence**  *Facilitators of adherence*  Personalised support from a credible healthcare professional. Individualised, tailored programme. Recording adherence and looking back at previous weeks to self-assess and monitor their progress was motivating. Intervention booklet supported adherence. |
| Minnella  2021 [78]  Canada | To assess the safety and the feasibility of personalized, stepped-approach prehabilitation care in the context of ERP for elective pulmonary cancer surgery. | **Access**  *Facilitators of access*  Physician referral. Residing in metropolitan area. Length of time to surgery.  *Barriers to access*  Travel distance to hospital.  **Adherence**  *Barriers to adherence*  Deteriorating health. Disinterest. |
| Moorthy  2023 [79] | To establish the feasibility of delivering a digital prehabilitation service. | **Access**  *Facilitators of access*  Direct referral.  **Adherence**  Adherence to the weekly touchpoints was 86%. In total, 100% of patients attended the T1 assessment and 55% attended the T2 session. The median time in the programme was 12 weeks. Twenty-six patients started the in-person service. Twenty-one patients were direct referrals to the service. Two patients (10%), both of whom had also declined the digital service, withdrew after a median of 4.5 weeks from commencing the programme. Average compliance to weekly touchpoints was 71%. Median time in the programme was 10 weeks.  *Facilitators of adherence*  Professional support across the 12-week programme and digital training. Training manual supplied. Option of one-on-one training with a digital care navigator. Clinical endorsement and support.  *Barriers to adherence*  Unfamiliar with English language, low digital literacy, feeling overwhelmed, limited access to mobile technology and technological issues, conflicting clinic appointments, rescheduling of surgery. |
| Murdoch  2021 [41] | To identify recommendations for improving intervention delivery within the main trial | **Access**  *Facilitators of access*  Healthcare professionals with sophisticated communication skills including the ability to use motivational interviewing.  *Barriers to access*  Structure and organisation of pre operative assessment; absence of information about importance of preoperative exercise; insufficient encouragement/motivation by staff to patients to exercise preoperatively; short lead in time to surgery. Insufficient motivation. Insufficient specialist prehabilitation staff capacity and space.  **Adherence**  In the hospital-supervised group 57% attended ≥6 pre-operative sessions. In the home supported group 70% participated in ≥2 telephone support sessions in the pre-operative phase.  *Facilitators of adherence*  Supportive, open relationships with patients motivated patients. Flexible, individualised support according to prior motivation and exercise levels.  *Barriers to adherence*  Limited availability of exercise bikes.  **Acceptance**  Patients responded positively to the exercise interventions, and some took up new activities, which suggests a good level of acceptability |
| Naito  2019 [80] | To test the feasibility of the early induction of new multimodal interventions specific for elderly patients with advanced non-small cell or pancreatic cancer | **Adherence**  Twenty-nine patients attended more than four of six planned sessions. The attendance rate was 96.7% (95% CI, 83.3 to 99.4). The median proportion of days wherein patients performed full or self-modified exercise programmes was 91%. Patients were compliant with consuming supplements, completing diaries, and wearing pedometers/ accelerometers. Most were also adherent to meeting the recommended nutritional requirements, continuing home-based exercise, and keeping or increasing their indoor or outdoor activities.  *Barriers to adherence*  Lack of motivation and feeling unwell. |
| Paynter  2017 [81] | To determine patient acceptance of a pre-operative immunonutrition supplement protocol and to compare post-operative outcomes pre- and post-implementation of the protocol | **Access**  *Barriers to access*  Timing of attendance to the pre-assessment clinic where the intervention was delivered. Late attendance meant it was too late to enable the full immunonutrition dose.  **Adherence**  *Facilitators of adherence*  Awareness of potential benefits of immunonutrition in terms of recovery from surgery.  *Barriers to adherence*  Dislike of taste or texture. Volume of supplement problematical.  **Acceptance**  Most found immunonutrition acceptable**.** |
| Piraux  2020 [82] | To assess the feasibility and the preliminary effects of a tele-prehabilitation program in esophagogastric cancer patients requiring surgery. | **Access**  *Facilitators of access*  Home-based prehabilitation with telecommunication support reduced transport. requirements.  **Adherence**  Of the 237 scheduled aerobic and resistance training sessions, 182 were completed, resulting in an overall attendance of 77%. Attendance at inspiratory muscle training was 68%, with 257 sessions completed of the 378 prescribed.  *Facilitators of adherence*  Exercise programmes tailored to the individuals physical fitness; weekly telephone calls provided motivational support and enabled programme adaptation if needed. Family support with website advice and support about the importance of preoperative exercise from the surgeon was also very helpful.  *Barriers to adherence*  Pain, insufficient motivation and energy, bad weather, disease progression, fatigue, no internet access, technical problems with the videos and the website interface unsuitable for digitally naïve participants.  **Acceptance**  The intervention was acceptable with satisfaction demonstrated across physiotherapist availability, the information brochure, programme intensity and duration, motivation, progress and participation. |
| Provan  2022 [63] | To document current practice, barriers and challenges to implementing prehabilitation in order to provide insight for the development of national frameworks for action and co-ordinated evaluation procedures in Scotland. | **Access**  *Barriers to access*  Insufficient understanding regarding prehabilitation and the core components of multi-modal prehabilitation, particularly as compared with ERAS. Insufficient awareness of local prehab provision. Short time frames between referral to treatment targets. Suboptimal human resources. Insufficient guidance and funding. Inequity in service provision. Patients reluctant to engage due to feeling unwell and need to attend multiple appointments. Financial costs. Patient isolation, poverty and lack of transport and broadband in rural and remote areas. Perception of an insufficient evidence base for prehabilitation. |
| Qin  2022 [83] | What is the association between health literacy and enhanced recovery after surgery (ERAS) adherence and postoperative outcomes in patients undergoing colorectal surgery? | **Access**  *Barriers to access*  Low health literacy  **Adherence**  *Facilitators of adherence*  High health literacy.  *Barriers to adherence*  Low health literacy. |
| Robinson  2023 [42] | 1. To identify frontline cancer HCP’ current views about physical activity for PABC relevant to their professional role and workplace  2. To determine if and when physical activity advice is currently part of usual practice amongst frontline cancer HCPs  3. To understand barriers and opportunities to integrating PA-based cancer rehabilitation within a range of cancer specific services, as well as eliciting HCP suggestions for future practice | **Access**  *Facilitators of access*  Physical activity as a priority for action. Early introduction to prehabilitation following diagnosis. Structured programmes to target lifestyles barriers. Good mental health was perceived as crucial to positively influence patient motivation and engage following diagnosis.  *Barriers to access*  Healthcare professionals perceived that physical activity rehabilitation advice and initiatives across cancer specialties are delivered on a non-systematic basis and service delivery is not joined up. Information about prehabilitation is fragmented, there is insufficient awareness among healthcare professionals about national recommendations for physical activity among adults, disagreement with regard who was responsible to discuss physical activity with patients and low levels of confidence to discuss physical activity with patients. Families can be over protective. Inequitable access and insufficient dedicated specialist space for rehabilitation. Patients lifestyles. Insufficient specialist staff and services to deliver programmes. Insufficient ownership and action around physical activity promotion.  **Adherence**  *Facilitators of adherence*  Positive mental health, engaging patients support network and enabling families to understand that physical activity is safe and appropriate. Healthcare professionals’ confidence with patient education, behaviour change techniques and motivational interviewing.  *Barriers to adherence*  Patients experiencing physical effects of disease and treatments and also intensive treatment regimes. Time constraints, costs, resources and staffing. |
| Rupnik  2020 [84] | To investigate the feasibility and safety of a multimodal intervention programme with partially supervised exercise training combined with nutritional support prior to HSCT. | **Access**  *Facilitator of access*  Haematopoietic stem cell transplantation (HSCT) candidate and donor availability.  *Barriers to access*  Musculoskeletal disorders restricting physical activity, high risk of fracture due to osteolytic bone lesions, uncontrolled pain, unstable ischaemic heart disease, severe chronic obstructive pulmonary disease, exertional asthma and a known allergy to whey protein.  **Adherence**  Patients performed the programme for an average of 6.8 weeks (median, 6.0; range, 2–14). The rate of participation in guided exercises averaged 73%, with at least two-thirds participation among 68% of patients. Diary data indicated that patients performed aerobic exercises for an average of 4.5 days per week and for an average duration of 132 min per week. A total of 67% of patients adhered to the aerobic exercise schedule at least 4 days per week, and 71% adhered to the aerobic exercise duration of at least 100 min per week. Patients performed the strength exercises on average 3.0 times per week. Seventy-eight percent of patients performed strength exercises at least 2.5 times per week. According to the sports bracelet data, patients took an average of 7416 steps per day and had at least 45 min of moderately intense physical activity (≥3 METs) per day. Eighty percent of participants completed the nutritional support plan and consumed at least 67% of the prescribed amount of protein powder.  *Barriers to adherence*  HSCT brought forward; onset of cardiac problems; early satiety, bloating and gastric discomfort and diarrhoea with nutritional supplements.  **Acceptance:**  Protein supplements generally well tolerated. |
| Santa Mina  2018 [85] | To assess the feasibility and effect of a personalised, home-based prehabilitation intervention on clinically-relevant outcomes in radical prostatectomy patients. | **Access**  *Facilitators of access*  Home-based intervention.  **Adherence**  Twenty-seven of 38 (69.2%) PREHAB participants met the minimum requirements of their individualized, home-based exercise protocol.  *Facilitators of adherence*  Home based intervention. Opportunity to request support.  *Barriers to adherence*  Exercise-induced pain, disinterest, surgery cancelled. |
| Shukla  2020 [86] | To determine the acceptability and perceived benefit of prehabilitation in lung cancer among thoracic surgeons | **Access**  *Barriers to access*  Surgeons perceived that access to AHP's/prehabilitation was not available. Need for urgent surgery or short time interval time for surgery. Patient comorbidities, referral difficulties, costs and transportation issues. |
| Solheim  2017 [87] | To assess the feasibility and potential efficacy of a multimodal intervention to attenuate cachexia in patients with incurable lung or pancreatic cancer | **Adherence**  Compliance was 76% (19/25) for the celecoxib, 60% (15/25) for the exercise components and 48% (12/25) for the nutritional supplement. Acceptable compliance was achieved in all but the nutritional supplement. Three patients had >80% compliance to all components of the intervention. In terms of combinations, eight (38%) patients did >80% of the aerobic and resistance components. Nine (43%) patients took >80% of the ONS and celecoxib components and nine (43%) patients took/did >80% of the resistance and celecoxib components.  *Barriers to adherence*  Hospitalisation, fatigue, insufficient time. Nutritional supplements perceived as unpalatable. |
| Stalsberg  2022 [50] | To investigate adherence to an outdoor 12-month post-surgery supervised exercise intervention during seasonal variation among newly diagnosed breast cancer patients receiving adjuvant treatment, and to identify sociodemographic and health-related adherence predictors. | **Access**  *Facilitators of access*  Ease of travel for prehabilitation, higher socioeconomic status , low risk of infectious diseases (outdoors), lower BMI and higher VO2max.  *Barriers to access*  Transport difficulties, costs and travel distance; workplace constraints and perceived burden of participating in group exercise.  **Adherence**  Mean adherence was 81% (median = 85.4) among completers (n = 36) and 63% (median = 78.8%) for the total intervention group (n = 47).  *Facilitators of adherence*  Staff presence during assessment, exercise and follow up. Mutual support between exercise group members.  *Barriers to adherence*  National holidays, age, comorbidities, travel, work and time constraints, health problems, exercise perceived as burdensome, family members’ health and needs, lower socio economic status, lower levels of education. |
| Steffens  2021 [51] | To establish the feasibility and acceptability of a preoperative exercise program, and to obtain pilot data on the likely difference in key surgical outcomes to inform the sample size calculation for a full-scale trial. | **Access**  *Facilitators of access*  Surgeons’ advice, support of family members.  **Adherence**  Of the 11 participants in the intervention group, the mean number of sessions per participant was 4: with participants attending 93% of the total face-to-face sessions (38 out 41). Of the 475 sessions that participants were advised to perform at home (171 home exercises and 304 daily physical activity advices), 302 (64%) were completed.  *Facilitators of adherence*  Recommended home exercises were easy to understand and follow. Duration and intensity of exercises was acceptable and addressed individual needs. Able to attend hospital for in person sessions.  **Acceptance**  Most participants were satisfied with the advice given by the treating physiotherapist, the amount and intensity of exercise given, and had no problems in coming to the hospital for weekly face to-face sessions. Participants were either satisfied or extremely satisfied with the preoperative exercise program, with all providing positive feedback about the pilot intervention: “It helped me manage moving; getting in and out of my hospital bed. I felt stronger and more confident to move after the operation”. None thought that the preoperative exercise program negatively affected them. |
| Sun  2020 [43]  United States | To determine the barriers and facilitators of adherence to a perioperative physical activity intervention in older adults with lung and gastrointestinal (GI) cancers and their family caregivers | **Adherence**  *Facilitators of adherence*  Routine physical activity was routine and included attending a gym, weight-lifting, hiking, yoga, and using resistance bands, regular participation in sporting activities. Walking with friends was a motivator. Using coping strategies including social activities, distraction, religious and faith groups and mind-body approaches, for example meditation to reduce stress. Goal setting was a strong motivator for physical activity engagement. Support from families by encouraging and participating in in physical activity intervention. Noticing the benefits of walking in terms of symptom reduction, anxiety, stress and relaxation. Use of assistive devices, such as single point cane, allowed some patients to ambulate for short distances.  *Barriers to adherence*  Comorbidities, health conditions and physical symptoms: chronic and treatment related pain, fatigue, peripheral neuropathy, nausea, insomnia, general weakness, recent surgery, injuries, medication sensitivity and allergies limited individuals’ ability to engage in physical activity. Functional limitations and a history of falls: difficulty with activities of daily living, gait and balance issues. Anxiety, feeling overwhelmed, psychological distress, stress. Roles, responsibilities and unexpected life events: primary caregiver for spouses with medical problems, full-time employment. Competing priorities: travel, lack of time and motivation, preference for sedentary lifestyle, disinterest, dislike of structured programs, laziness, not accustomed to physical activity and dislike of walking. Physical environment and weather. |
| Thoft Jensen 2019 [52] | To assess feasibility of an existing Danish home-based prehabilitation program when implemented in a US cancer centre | **Adherence**  The majority of patients demonstrated adherence to the prehabilitation program.  Twenty patients (62.5%) were adherent to the exercise component of the prehabilitation program. Adherence to the nutritional component was 81.2%.  *Barriers to adherence*  Neoadjuvant chemotherapy was a predictor of non-adherence.  **Acceptance**  Authors claimed that a short-term multimodal prehabilitation program, prior to major bladder cancer surgery in a comprehensive cancer centre, was feasible and well-accepted. |
| Tweed  2021 [53] | To explore the feasibility of the BEFORE (Better Exercise  and Food, Better Recovery) multimodal prehabilitation program consisting of personalized, ambulatory, hospital based exercise training, and fresh protein-rich food in terms of compliance, organization and acceptance to outline the design of a large, statistically well-powered comparative trial. | **Access**  *Barriers to access*  Previous surgical history, informal caring responsibilities, other surgery scheduled, dietary restrictions, travel difficulties, lived too far away from the hospital, programme considered too demanding.  **Adherence**  Seven patients (77.8%) attended ≥80% of all 12 training. All patients (100%) attended more than 80% of the available training sessions. Individual attendance to the 12 training sessions ranged from 75.0–95.8% with a median of 91.7%, and 2 patients (22.2%) attended all sessions. Six patients (66.7%) consumed more than 80% of the required protein intake, and all participants (100%) consumed more than 80% of the required energy intake. The median percentage of consumed meals and snacks was 95.2% (IQR 79.8–97.6%), and one patient consumed all meals and snacks.  *Barriers to adherence*  Surgery brought forward.  **Acceptance**  Participants considered the interventions to be acceptable in terms of frequency and intensity of physical training, and regarding number and taste of the meals. The training program was appraised as good or excellent by all patients; most patients rated the intensity as sufficient (n = 4, 44.4%) or heavy (n = 4, 44.4%). The number of training sessions was appropriate (n = 9, 100%), and the length of sessions was sufficient (n = 7, 77.8%). Generally, patients accepted the extra hospital visits (n = 5, 55.5%) but four patients considered the extra visits burdensome. Most patients had a positive food experience; 77.8% rated the taste and quality as good or excellent. The number of meals was considered a lot (n = 6, 66.7%) or too much (n = 2, 22.2%), and meal portions were considered sufficient (n = 7, 77.8%). |
| Van Rooijen  2019 [54] | To test the feasibility, safety, and effectiveness of a multimodal prehabilitation program intended to be studied in a randomized controlled trial | **Access:**  *Facilitators of access*  Surgeons recommendation, desire to be more active and prepare self for treatment.  **Adherence**  Attendance at training sessions was high: of the 17 (85%) participants who completed the program, 12 (71%) attended 90% or more of the 12 intended training sessions. The remaining 5 (29%) also attended more than 75% of training sessions, and overall, 88% of training sessions were completed. All scheduled visits to the dietitian and psychologist were attended.  *Facilitators of adherence*  Professional support, sense of enhancing health, prehabilitation was a distraction and offered participants a sense of control,  *Barriers to adherence*  Informal caregiving responsibilities, insufficient time and surgery brought forward. Endurance and resistance training, following dietary advice and protein supplementation and psychological support were challenging. Protein consumption just before sleep caused discomfort and nausea.  **Acceptance**  The programme was acceptable to participants. When asked if they would follow the program again in retrospect, all patients confirmed, and all patients would recommend the program to family or friends. |
| Waller  2022 [55] | To assess the efficacy of a tri-modal prehabilitation programme delivered by smartwatches for improving functional fitness prior to major abdominal cancer surgery. | **Access**  *Barriers to access*  Disinterest, too far to travel, other commitments.  Fit bit and smartphone based intervention worked well in our smartphone age.  **Adherence**  Prehabilitation group participants wore the Fitbit Charge 2 on 98.9% of days. Compliance with the exercise component was high, with participants achieving 30 min of moderate-intensity activity on average 59.9% (95% CI, 41.3–78.5) of the days during the pre-operative period. Compliance to resistance training component could not be objectively monitored via the Fitbit. Usability of the Fitbit food log was high, with participants logging food on average 82.9% of the days. Participants on average only listened to the mindfulness App on 15% of days.  *Facilitators of adherence*  Smartphone app and wearable technology motivated participants. Standardised weekly phone calls allowed reporting of technical issues and provided tailored prehabilitation support.  *Barriers to adherence*  The wellbeing application was considered as being superfluous to individual needs. Reasons for non-adherence to the mindfulness intervention included participants reporting good baseline mental health, finding the app unhelpful, and only using the app when they felt they needed to. Technical issues were experience with the Fitbit. Adherence to protein targets was difficult.  **Acceptance**  All participants rated both the prehabilitation programme overall and the exercise component “Good” or “Excellent”. Ten participants (90%) responded “Strongly Agree” or “Agree” to the statement “The Fitbit motivated me to do the physical activity that was part of the prehabilitation programme”. |
| Waterland  2021 [64] | To evaluate the current and likely future impact of a telehealth preoperative education package for patients preparing for major abdominal cancer surgery | **Access**  *Facilitators of access*  Home based. Online delivery eliminated participants need to travel, pay for parking and wait around at the hospital.  *Barriers to access*  Forgetfulness, technological difficulties, previous experience of surgery, work commitments, insufficient time prior to surgery and feeling overwhelmed.  **Adherence**  *Barriers to adherence*  Duplication of information previously received.  **Acceptance**  Most (31; 97%) participants who attended the online education session reported they would recommend it to others preparing for surgery. Of participants who attended the webinar, 24 (77%) reported if given a choice they would attend the online education session as opposed to attending the hospital based session. |
| Waterland  2022 [56] | To investigate the feasibility of delivering a hospital- and community-based prehabilitation program in patients identified at high risk of postoperative complications | **Access**  *Facilitators of access*  Home based prehabilitation.  *Barriers to access*  Distance to hospital, feeling overwhelmed, exercise programme already in place.  **Adherence**  Of the 50 participants who consented to participate, 44 (88%) patients attended their exercise prescription appointment, 36 (72%) attended their respiratory exercise education session, 43 (86%) completed repeat functional assessments (6MWT and handgrip strength), 42 (84%) had repeat CPET testing, and 35 (70%) proceeded to their planned surgery.  *Facilitators of adherence*  Home based resistance exercise.  *Barriers to adherence*  Fatigue, conflicting appointments, traveling for appointments, busy with other commitments, for example family illnesses), illness, for example a common cold, pain, weather, boredom, and poor motivation when setbacks occurred, for example surgery date postponed. |
| Wu  2021 [57] | To determine the feasibility of multimodal prehabilitation as part of the breast cancer treatment pathway | **Access**  *Barriers to access*  Imminent surgery; full time work and/or caring commitments; transport difficulties; disinterest; scheduled appointments.  **Adherence**  *Barriers to adherence*  Physical discomfort (muscle soreness) and lack of interest.  **Acceptance**  Twenty five (93%) patients regarded the service as excellent and a powerful motivator for achieving healthy lifestyle changes. They perceived the service to be beneficial, as part of their cancer treatment, their postoperative recovery, and they would strongly recommend it to other patients. |
| Wu  2022 [44] | To describe our patients' perceptions of tele-prehabilitation and capture their capabilities, opportunities, and motivations to participate. | **Access**  *Facilitators of access*  Healthcare professionals and other people affected by cancer who shared information about the service. The way in which prehabilitation information was provided by healthcare professionals facilitated access. Telehealth enabled prehabilitation access when typically, it would geographically fall outside of participants’ catchment area. Health concerns as a result of the cancer diagnosis activated participants to seek support from prehabilitation service and motivated them to adopt healthier lifestyles, improve health outcomes and recovery post treatment, reduce the risk of chronic illness and improve their quality of life.  *Barriers to access*  Insufficient awareness of prehabilitation programme and local support available prior to cancer diagnosis.  **Adherence**  *Facilitators of adherence*  Regular, positive interactions with and support from prehabilitation team, particularly during Covid-19. The counselling component psychologically prepared participants to engage with prehabilitation. A positive mindset was a motivator for action and engagement with prehabilitation to cope with their disease and recovery. Personalised prehabilitation tailored to individual needs and goals and where progress was tracked. Goal attainment was motivational. Encouragement and support from family and friends motivated sustained adherence. Family members would also engage in the programme. Flexibility and convenience of a home-based programme delivered remotely and was less stressful and anxiety provoking than hospital visits. Ability to incorporate exercise into daily routines, including work and personal commitments.  **Acceptance**  Participants welcomed the flexibility of the tele-prehabilitation service. They did not need to travel, enabling those with a lack of access to hospital transport or those living outside of the region to participate. They were able to engage in the intervention in the safety and comfort of their own home, which may have facilitated engagement for some participants with physical limitations or those experiencing hospital anxiety. One disadvantage was the missed opportunity for peer support. |
