# Supplementary material for: Access, acceptance and adherence to cancer prehabilitation: a mixed-methods systematic review
Source: J Cancer Surviv. 2024 May 6;19(6):1895–923. doi: 10.1007/s11764-024-01605-3 (PMC12546383; doi:10.1007/s11764-024-01605-3)
Supplement: Supplementary file 3 — Supplementary file3 (DOCX 42.6 KB) [file 11764_2024_1605_MOESM3_ESM.docx]

| **Study author**  **Year** | **Type of Study** | **Screening questions**  **S1 S2** | **Qualitative studies**   - 1. **1.2 1.3 1.4 1.5** | **Randomized Controlled Trials**  **2.1 2.2 2.3 2.4 2.5** | **Non-Randomized studies**  **3.1 3.2 3.3 3.4 3.5** | **Quantitative descriptive studies**  **4.1 4.2 4.3 4.4 4.5** | **Mixed Methods studies**  **5.1 5.2 5.3 5.4 5.5** |
| --- | --- | --- | --- | --- | --- | --- | --- |
| Prepare ABC Trial Collaborative 2021  [28] | Quantitative | Y Y |  | Y Y Y Y C |  |  |  |
| Agasi-Idenburg 2020 [32] | Qualitative | Y Y | Y N Y Y Y |  |  |  |  |
| Argudo 2021  [29] | Quantitative | Y Y |  |  | Y Y Y Y Y |  |  |
| Banerjee  2019 [33] | Qualitative | Y Y | Y Y Y Y Y |  |  |  |  |
| Beck  2021 [45] | Mixed Methods | Y Y | Y Y Y Y Y |  |  | CT CT Y CT CT | N N N CT N |
| Beck  2022 [34] | Qualitative | Y Y | Y Y Y Y Y |  |  |  |  |
| Beck  2021 [46] | Mixed Methods | Y Y | Y Y Y Y Y |  |  | Y Y Y CT CT | CT CT Y CT Y |
| Bingham  2023 [12] | Qualitative | Y Y | Y Y Y Y Y |  |  |  |  |
| Bradley  2023 [13] | Quantitative | Y Y |  |  |  | Y Y CT Y Y |  |
| Brady  2020 [35] | Qualitative | Y Y | Y Y Y Y Y |  |  |  |  |
| Brahmbhatt  2020 [58] | Mixed Methods | Y Y | Y Y Y Y Y |  | Y Y N N Y |  | N Y Y N N |
| Burden  2017 [30] | Quantitative | Y Y |  | Y Y CT Y Y |  |  |  |
| Catho  2021 [31] | Quantitative | Y Y |  |  |  | Y Y Y N Y |  |
| Collaco  2021 [36] | Qualitative | Y Y | Y Y Y N Y |  |  |  |  |
| Cooper  2022 [37] | Qualitative | Y Y | Y Y Y Y Y |  |  |  |  |
| Crowe  2022 [47] | Quantitative | Y Y |  |  |  | Y Y Y N Y |  |
| Daun  2022 [38] | Qualitative | Y Y | Y Y Y Y Y |  |  |  |  |
| Deftereos  2022 [59] | Mixed Methods | Y Y | Y Y Y Y Y |  |  | CT CT Y N Y | Y Y Y Y Y |
| Deftereos  2021 [48] | Quantitative | Y Y |  |  |  | CT Y Y Y Y |  |
| Drummond  2022 [49] | Quantitative | Y Y |  |  |  | Y Y Y CT Y |  |
| Ferreira  2018 [88] | Qualitative | Y Y | Y N N N N |  |  |  |  |
| Ferreira  2021 [72] | Quantitative | Y Y |  | Y Y Y CT Y |  |  |  |
| Franssen  2022 [73] | Quantitative | Y Y |  |  |  | Y N Y N Y |  |
| Halliday  2021 [74] | Quantitative | Y Y |  |  |  | Y Y Y CT Y |  |
| Hogan  2019 [39] | Qualitative | Y Y | Y N N N N |  |  |  |  |
| Karlsson  2019 [75] | Quantitative | Y Y |  | Y N Y Y Y |  |  |  |
| Lawson  2021 [76] | Quantitative | Y Y |  | Y N Y N Y |  |  |  |
| Low  2020 [60] | Mixed Methods | Y Y | Y N N N N |  | CT N CT CT N |  | Y N N N N |
| Machado  2023 [77] | Quantitative | Y Y |  |  |  | Y CT Y CT Y |  |
| Macleod  2018 [61] | Mixed Methods | Y Y | Y Y CT N N |  | CT Y N N N |  | Y N N CT N |
| Mawson  2021 [62] | Mixed Methods | Y Y | Y Y CT N CT |  |  | CT CT Y N N | Y N N N N |
| McCourt  2023 [40] | Qualitative | Y Y | Y Y Y Y Y |  |  |  |  |
| Minnella  2021 [78]  Canada | Quantitative | Y Y |  |  | Y Y Y N CT |  |  |
| Moorthy  2023 [79] | Quantitative | Y Y |  |  |  | N N Y N Y |  |
| Murdoch  2021 [41] | Qualitative | Y Y | Y Y Y Y Y |  |  |  |  |
| Naito  2019 [80] | Quantitative | Y Y |  |  |  | Y Y Y N Y |  |
| Paynter  2017 [81] | Quantitative | Y Y |  |  |  | Y Y Y N Y |  |
| Piraux  2020 [82] | Quantitative | Y Y |  |  |  | N N Y Y Y |  |
| Provan  2022 [63] | Mixed Methods | Y Y | Y Y CT CT Y |  |  | Y CT CT Y N | Y N N N N |
| Qin  2022 [83] | Quantitative | Y Y |  |  | Y Y CT Y Y |  |  |
| Robinson  2023 [42] | Qualitative | Y Y | Y Y Y Y Y |  |  |  |  |
| Rupnik  2020 [84] | Quantitative | Y Y |  |  |  | Y Y Y N Y |  |
| Santa Mina  2018 [85] | Quantitative | Y Y |  | CT N N CT Y |  |  |  |
| Shukla  2020 [86] | Quantitative | Y Y |  |  |  | Y Y N N N |  |
| Solheim  2017 [51] | Quantitative | Y Y |  |  |  | Y CT Y Y Y |  |
| Stalsberg  2022 [50] | Quantitative | Y Y |  |  | N Y Y Y Y |  |  |
| Steffens  2021 [51] | Quantitative | Y Y |  | Y Y Y Y Y |  |  |  |
| Sun  2020 [43]  United States | Qualitative | Y Y | Y N Y Y N |  |  |  |  |
| Thoft Jensen 2019 [52] | Quantitative | Y Y |  |  |  | Y Y Y CT Y |  |
| Tweed  2021 [53] | Quantitative | Y Y |  |  |  | Y Y Y N Y |  |
| Van Rooijen  2019 [54] | Quantitative | Y Y |  |  | Y Y Y N CT |  |  |
| Waller  2022 [55] | Quantitative | Y Y |  | Y Y Y CT Y |  |  |  |
| Waterland  2021 [64] | Mixed Methods | Y Y | Y N Y Y Y |  |  | CT CT Y N CT | Y Y N N N |
| Waterland  2022 [56] | Quantitative | Y Y |  |  |  | Y Y Y CT Y |  |
| Wu  2021 [57] | Quantitative | Y Y |  |  | Y Y CT CT Y |  |  |
| Wu  2022 [44] | Qualitative | Y Y | Y Y Y Y Y |  |  |  |  |

Abbreviations: Y = Yes; N = No; CT = Cannot tell

**Screening questions**

S1. Are there clear research questions?

S2. Do the collected data allow to address the research questions?

**Methodological quality criteria**

1. **Qualitative**

1.1. Is the qualitative approach appropriate to answer the research question?

1.2. Are the qualitative data collection methods adequate to address the research question?

1.3. Are the findings adequately derived from the data?

1.4. Is the interpretation of results sufficiently substantiated by data?

1.5. Is there coherence between qualitative data sources, collection, analysis and interpretation?

1. **Quantitative randomized controlled trials**
   1. Is randomization appropriately performed?
   2. Are the groups comparable at baseline?
   3. Are there complete outcome data?
   4. Are outcome assessors blinded to the intervention provided
   5. Did the participants adhere to the assigned intervention?
2. **Quantitative non-randomized**

3.1. Are the participants representative of the target population?

3.2. Are measurements appropriate regarding both the outcome and intervention (or exposure)?

3.3. Are there complete outcome data?

3.4. Are the confounders accounted for in the design and analysis?

3.5. During the study period, is the intervention administered (or exposure occurred)

1. **Quantitative descriptive**

4.1. Is the sampling strategy relevant to address the research question?

4.2. Is the sample representative of the target population?

4.3. Are the measurements appropriate?

4.4. Is the risk of nonresponse bias low?

4.5. Is the statistical analysis appropriate to answer the research question?

1. **Mixed methods**

5.1. Is there an adequate rationale for using a mixed methods design to address the research question?

5.2. Are the different components of the study effectively integrated to answer the research question?

5.3. Are the outputs of the integration of qualitative and quantitative components adequately interpreted?

5.4. Are divergences and inconsistencies between quantitative and qualitative results adequately addressed?

5.5. Do the different components of the study adhere to the quality criteria of each tradition of the methods involved?
